# Effective Ciprofloxacin Removal from Deionized and Salt Water by Sulfonated Pentablock Copolymer (Nexar^TM^)

**DOI:** 10.3390/molecules30153275

**Published:** 2025-08-05

**Authors:** Simona Filice, Simona Crispi, Viviana Scuderi, Daniela Iannazzo, Consuelo Celesti, Silvia Scalese

**Affiliations:** 1 Consiglio Nazionale delle Ricerche, Istituto per la Microelettronica e Microsistemi (CNR-IMM), Ottava Strada n.5, I-95121 Catania, Italy; simona.crispi@cnr.it (S.C.); viviana.scuderi@imm.cnr.it (V.S.); 2 Department of Engineering, University of Messina, Contrada Di Dio, I-98166 Messina, Italy; daniela.iannazzo@unime.it (D.I.); consuelo.celesti@unime.it (C.C.)

**Keywords:** pentablock copolymer, adsorption, ciprofloxacin antibiotic, water treatment, salt water

## Abstract

The presence of ciprofloxacin antibiotic in water is a threat to humans and aquatic life since antibiotics are currently regarded as emerging contaminants of major concern. This work reported the use of Nexar^TM^ film, a sulfonated pentablock copolymer, to effectively remove ciprofloxacin antibiotic from water in a sustainable approach. The removal efficiency of Nexar film was evaluated in aqueous or salty (NaCl 0.5 M) ciprofloxacin solutions as a function of contact time and the initial ciprofloxacin concentration. In the investigated conditions, the polymeric film totally removed ciprofloxacin in MilliQ solution while its removal efficiency in salty solution was approximately 73%. This lower value is due to the presence of Na^+^ ions that compete with antibiotic molecules for adsorption on active surface sites of the polymeric film. No further release of adsorbed antibiotic molecules occurred. The kinetic studies, conducted for ciprofloxacin adsorption on Nexar film in both MilliQ and salty solutions, revealed that the overall sorption process is controlled by the rate of surface reaction between ciprofloxacin molecules and active sites on Nexar surface. Furthermore, at equilibrium conditions, the isotherm model that best fits experimental parameters was not linear. This indicates that the competition between the solute and the solvent for binding sites on the adsorbent should be considered to describe adsorption processes in both MilliQ and salty solutions.

## 1. Introduction

Since the discovery of penicillium, the use of antibiotics has growing constantly to treat infectious diseases like the plague, cholera, pneumonia, tuberculosis, diarrhea, gonorrhea, syphilis, influenza, etc. that were the major health concerns during the 20th century [1]. Furthermore, antibiotics also found application in veterinary uses as supplements in animal feed [2,3] and in the agrifood sector where the practice of adding antibiotics in water used for irrigation mixed to fertilizers and pesticides has increased considerably in order to enhance the yield [4].

Due to the extensive and inappropriate usage, antibiotics accumulate in the soil and water sources affecting the health of ecosystems and humans by reducing microbial diversity [5], increasing the presence of resistance genes [6], and potentially impacting the health of fish [7] and algae [8]. The main concerns regarding human health are the development of allergic reactions, toxicity, and neurological and psychiatric issues in the case of some antibiotics like fluoroquinolones and sulfonamides [3]. Of particular interest is the development of antibiotic resistance in microorganisms caused by antibiotic overuse and pollution, which is making it difficult to treat a growing number of infections [9]. The World Health Organization (WHO) has recognized antimicrobial resistance (AMR) as a critical global threat, with projections suggesting that resistant bacterial infections could become the leading cause of global deaths by 2050 [10].

Besides the lack of proper regulations of pharmaceutical disposal in many countries and the lack of sensitive detection methods of drug presences in the ecosystem [11], there is growing evidence that the development of new antibiotic removal technologies being implemented within existent water treatment methodologies is urgently needed in order to find a solution to counter the threat posed by antibiotic accumulation in the ecosystem [12]. Indeed, approximately 95% of the antibiotics are expelled unmodified and every antibiotic drug inevitably ends up in the environment, particularly in water [13].

Ciprofloxacin (CIP) is a quinolone antibacterial agent commonly used to treat many types of infections caused by Gram-negative bacilli bacteria, some Gram-positive bacteria and Enterobacteriaceae such as pneumonia, diarrhea, infections of the skin, bone, joint, abdomen, prostate, sinus, kidney, urinary tract, and bronchitis [14]. It is toxic for marine environments and aquatic species: it causes morphological alterations in embryos which may lead to their death [15,16]. The removal of CIP from wastewater represents a great challenge [17] since it is highly stable and difficult to degrade due to the presence of fluorine ion in the CIP structure.

Conventional wastewater treatment plants are often not designed to completely remove pharmaceutical compounds [18]. Indeed, some advanced wastewater treatment methods can substantially decrease antibiotic residues [19] such as photodegradation [19,20], catalytic oxidation [21], membrane separation and adsorption [22], electro-Fenton process [23], and biodegradation [24]. However, such technologies have not been implemented, since they are expensive, inefficient, non-green, and impractical [25,26]; almost half of the world’s wastewater is released to the environment without any treatment [26]. As a result, treated wastewater can still contain these substances, which are then discharged into waterways.

Among water treatment technologies, adsorption by solid adsorbents has many advantages, including ease of the operation, high efficiency, and the possibility to be applied for the removal of different types of pollutants [16,27,28]. Furthermore, an extensive range of materials like gels, films, membranes, particles, etc., can be used as adsorbents, making it more relevant. The most investigated adsorbent materials are carbon-based materials [15] and activated carbon (ACs) [29] that can be used also as a replacement for anthracite media in conventional filters [30]. Nanostructured powders showed different values of adsorption capacities, ranging from some to hundreds milligrams of ciprofloxacin adsorbed per gram of powder [31]. Although their efficiency, the use and regeneration of above-mentioned powders is costly. Thus, the recent research has been devoted to the search for low-cost adsorbent materials with high efficiency and the possibility of being reused [32]. Over the last few years application of bio-based adsorbents formulated by using natural feedstock, i.e., biochar [33], natural and chemically modified clays [34], polymers, and biopolymers [35] have received great attention. In the field of polymeric materials, more studies are still needed on the improvement of polymeric adsorbent/filters selectivity toward specific pollutants, on their stability, filtration efficiency, and regeneration. Indeed, low removal efficiencies of antibiotics or pharmaceutical compounds have been achieved by commonly used ultrafiltration (UF), nano-filtration (NF), and reverse osmosis (RO) membranes in addition to other drawbacks, such as high-pressure requirement and membrane fouling [36]. Therefore, a more promising treatment method appears to be achieved by adsorptive membranes that can show a twofold function of adsorption and filtration processes [37] and are able to selectively remove water contaminants depending on specific chemical and/or physical interactions. Selectivity can be achieved in different ways, e.g., by dispersing in a polymeric matrix active nanofillers able to adsorb specific water contaminants such as organic dyes [38,39,40], pharmaceuticals [41], inorganic anions and cations [42], or by introducing active surface groups on the polymeric backbone.

In this work, we report the use of a sulfonated pentablock copolymer, commercially named Nexar^TM^ for the removal of ciprofloxacin from deionized and salt water, the latter being useful for assessing the polymer adsorption properties in sea water. Nexar^TM^ polymer is a symmetric pentablock copolymer comprised of poly[t-butyl styrene-b-hydrogenated isoprene-b-sulfonated styrene-b-hydrogenated isoprene-b-t-butyl styrene] (tBS-HI-SS-HI-tBS) [42]. Nexar^TM^ showed to be characterized by a good trade-off between permeability and stability, thanks to this specific polymeric architecture, in which the sulfonated midblock provides the ionic and acid character, while the outer blocks provide the flexibility of a low glass transition (Tg) material and the strength of a high Tg material. Its antimicrobial and antibiofouling properties [43,44,45,46] make it highly efficient in filtration processes [43,47,48]. Furthermore, it showed to be both a high capacity and selective adsorbent for organic and inorganic water contaminants and to be a synergic support for photocatalytic particles in water contaminants photodegradation processes [43]. The adsorption abilities of Nexar^TM^ for ciprofloxacin removal in water and salty solution are here reported for the first time. Chemical, thermal, and physical properties of the polymer were fully investigated and the ciprofloxacin removal mechanism by Nexar^TM^ film was studied through adsorption kinetic models and adsorption isotherms in both MilliQ and salt water solutions.

## 2. Results

### 2.1. Characterization of Membranes

The membrane morphology and thickness were analyzed by SEM, revealing a smooth and homogeneous surface and a thickness of 35 µm measured on the cross-section, as reported in Figure S1 of Supporting Information.

The FT-IR spectrum of Nexar film acquired in the region between 4000 and 500 cm^−1^ is reported in Figure S2 of Supporting Information.

IR peaks within 3100 and 3700 cm^−1^ are due to the stretching vibrations of the hydroxyl group (OH group) from the water absorbed. The large peak at 3056 cm^−1^ is characteristic of unsaturated systems, including alkenes (C=C bonds) and aromatic rings. The features between 2800 and 3000 cm^−1^ are generally associated with saturated systems (alkanes, sp^3^ hybridized carbon-hydrogen bonds). We attribute these peaks to the asymmetric and symmetric CH stretching vibrations of the methyl groups. The features in the region between 2400 cm^−1^ and 2000 cm^−1^ are strongly influenced by the presence of environmental carbon dioxide. The peak at 1640 cm^−1^ is related to C=C stretching. The small feature at 1595 cm^−1^ is due to aromatic hydrocarbons that exhibit absorptions in the 1600–1585 cm^−1^ region due to C-C stretching vibrations in the aromatic ring. The IR peak around 1452 cm^−1^ typically indicates the presence of methylene (CH_2_) or methyl (CH_3_) groups undergoing bending vibrations. The peaks at 1411 cm^−1^, 1160 cm^−1^, 1126 cm^−1^, 1035 cm^−1^, and 1006 cm^−1^ are due to SO_3_^−^ (characteristic peaks). Peaks at lower wavenumbers are related to epoxy at 823 cm^−1^, a peak around 699 cm^−1^ is characteristic of out-of-plane ring deformation vibrations of the phenyl group (C-H bonds in a benzene ring), often used to identify the presence of styrene or similar aromatic compounds.

**Figure 1:**
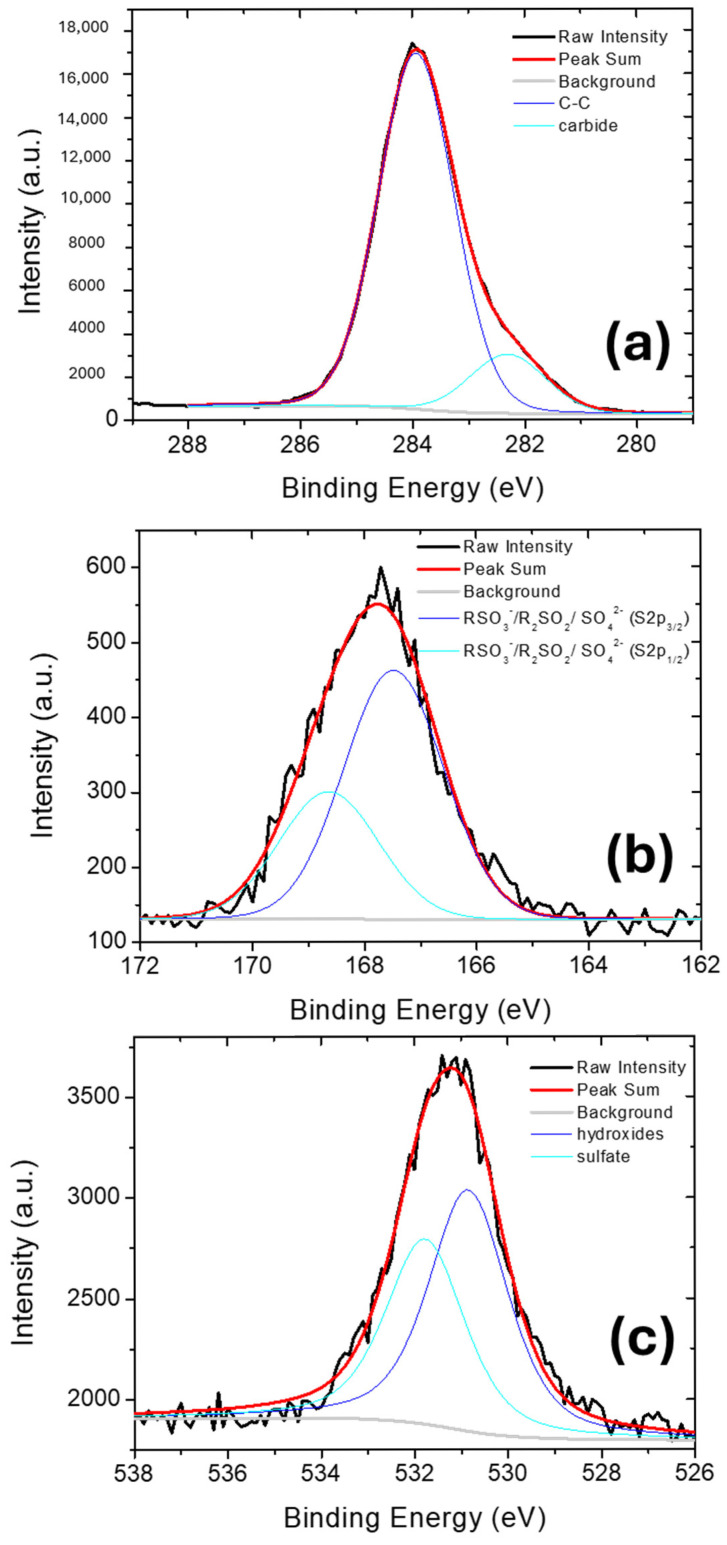
XPS spectra of C1s (**a**), S2p (**b**) and O1s (**c**) for Nexar film. Black continuous lines indicate the acquired spectra, red lines are the fits of the spectra, grey lines are the subtracted baselines, and the other lines are the contributions obtained by peak deconvolution.

The chemical composition of Nexar surface was investigated by XPS analysis. XPS survey spectrum is reported in Figure S3 of the Supporting Information and the main signals observed are related to sulfur (S2p at 168 eV), carbon (C1s at 284 eV) and oxygen (O1s at 530 eV) elements. Figure 1 reports deconvolutions of C1s, S2p, and O1s spectra, respectively, to study the chemical nature of each element present on film surface. By these deconvolutions, the relative amounts (%) of each species were estimated by the corresponding peak area and are reported in Table 1.

C1s spectrum of commercial Nexar film is deconvoluted into two peaks at 282.3 and 283.9 eV that are associated to carbide/vacancies and C-C bonds, respectively. We suppose the first peak is due to the synthetic procedure of polymeric backbone.

S2p peak contains two components at 167.5 eV and 168.6 eV due to spin-orbit splitting, i.e., S2p_3/2_ and S2p_1/2_, (energy difference 1.16 eV and intensity ratio of 0.511). It can be related to sulfur with high oxidation state, i.e., sulfonic acid group (RSO_3_^−^), sulfone, and sulfate assigned to S2p_3/2_ and S2p_1/2_, respectively [49]. The O1s spectrum can be deconvoluted into two peaks having binding energy at 530.8 and 531.8 eV, respectively. The first peak is assigned to hydroxides, i.e., O-H bond associated with sulfonic acid groups. The second peak is assigned to sulfate, i.e., O=S of SO_3_^−^ groups incorporated during the sulfonation of the polystyrene films [49]. These functionalities are responsible for imparting the hydrophilicity to the membranes.

**Figure 2:**
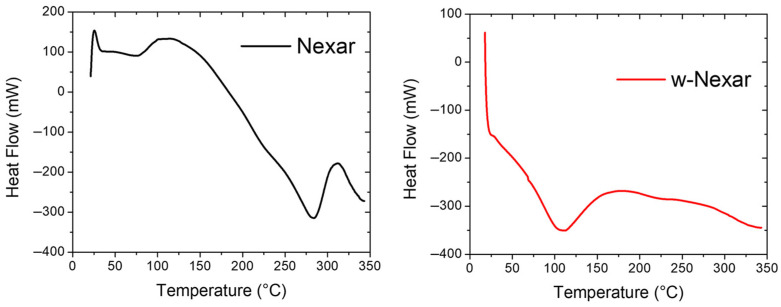
DSC thermograms of the Nexar films as received (**on the left**) and after washing (**on the right**). The experiments were performed under N_2_ atmosphere.

**Figure 3:**
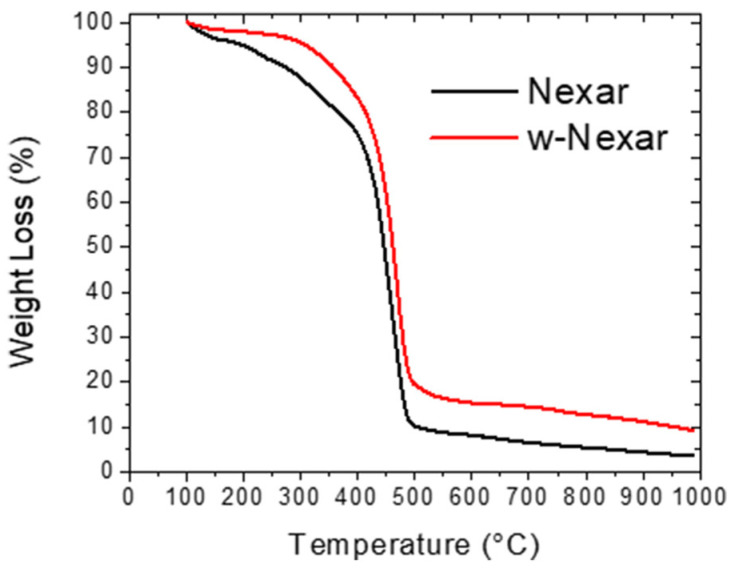
TGA profiles of Nexar films as received and after washing. The experiments were performed under Ar atmosphere.

Given the importance of thermal stability for the application of Nexar films in water purification processes, the thermal behavior of both Nexar and w-Nexar (washed Nexar as described in Section 3.2) was assessed using differential scanning calorimetry (DSC) and thermogravimetric analysis (TGA) reported in Figure 2 and Figure 3, respectively.

The DSC curve of Nexar revealed a glass transition (Tg) at approximately 10–20 °C, characterized by a subtle shift in the heat flow baseline, while w-Nexar showed a similar transition slightly shifted to lower temperatures. This decrease in Tg after washing suggests increased molecular mobility, attributed to the plasticizing effect of water molecules adsorbed during the washing process. These water molecules can interact with the sulfonic acid groups present in the polymer through hydrogen bonding, increasing the free volume and weakening interchain interactions, thereby facilitating segmental motion at lower temperatures. This is primarily due to the fact that water molecules adsorbed in the polymer can act as plasticizers, increasing the free volume and allowing the polymer chains to move more freely [50,51,52]. The swelling behavior of Nexar in water is well reported [53]. After immersion in MilliQ water, an 84% increase in film thickness (from 50 to 92 μm) along with a water uptake of 168% for the commercial Nexar, was observed. A broad endothermic transition was observed in Nexar between approximately 75 °C and 150 °C, which likely corresponds to the relaxation or partial breakdown of ionic domains or absorbed water loss. In w-Nexar, this transition appeared broader and more intense, starting from ~50 °C and extending up to ~200 °C, indicating a change in the polymer’s internal structure, such as reduced crystallinity and altered ionic clustering, caused by the washing treatment. At higher temperatures (270–320 °C), Nexar exhibited an exothermic peak, potentially related to the crystallization or reorganization of hydrophobic segments like alkyl side chains, a feature less evident or absent in the w-Nexar sample, likely due to prior structural changes. Overall, the DSC profiles clearly demonstrate that the washing process alters the thermal transitions and morphology of the Nexar film, primarily through water-induced plasticization and reconfiguration of its microstructure [50,51,52,53].

Figure 3 reports the TGA curves of both commercial and washed Nexar films.

Thermogravimetric analysis (TGA) of Nexar and w-Nexar films reveals three distinct regions of mass loss that reflect key changes in thermal stability. An initial weight loss below 150 °C (~10%) corresponds to the evaporation of physically adsorbed water, consistent with the hydrophilic nature of the sulfonic acid groups in the polymer. A major degradation step occurs between 260 °C and 400 °C, attributed to the desulfonation of the polymer backbone [40]. In this range, the w-Nexar sample exhibits a less pronounced and more gradual weight loss compared to Nexar, indicating enhanced thermal stability, likely due to the formation of hydrogen bonds of retained water molecules after washing. Beyond 400 °C, both samples continue to degrade slowly, but w-Nexar retains slightly more mass, suggesting increased resistance to thermal decomposition and possibly more stable char residue formation.

In conclusion, the surface of Nexar film is characterized by the presence of sulfonic acid groups that confer it high hydrophilicity. Furthermore, the pretreatment of the membrane with water affects its polymeric structure, improving its properties, i.e., higher resistance to thermal degradation and lowering its Tg consequently to the adsorption of water molecules.

### 2.2. Ciprofloxacin Adsorption Experiments

#### 2.2.1. Adsorption Processes in MilliQ Water or Salt Water

Ciprofloxacin adsorption experiments were performed as reported in Section 3.4. Before tests, an appropriate calibration curve from low to high concentrations of the antibiotic was performed to verify the linearity of the Beer–Lambert law within the investigated concentration range, i.e., from 1.15 to 28.75 ppm. UV-visible absorbance spectra of ciprofloxacin solutions at different concentrations and the relative calibration curve are reported in Figure S2 of the Supporting Information.

**Figure 4:**
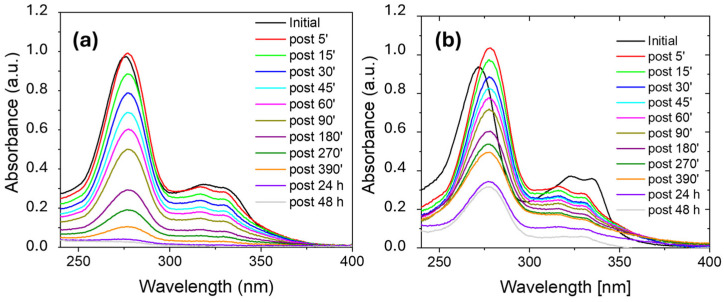
UV-visible absorbance spectra versus time obtained for Ciprofloxacin solutions prepared using MilliQ water (**a**) and salt water (**b**) where Nexar film was immersed. The initial concentration of Ciprofloxacin was 10 ppm.

Figure 4 reports the UV-visible absorbance spectra for ciprofloxacin aqueous solutions (10 ppm), where a 1 cm^2^ piece of Nexar was immersed, at different adsorption times. Ciprofloxacin removal efficiency was measured by acquiring the UV-Vis spectra as a function of time (see Figure 4a). This process was compared with the same process conducted in salt water (i.e., 0.5 M NaCl solution) as reported in Figure 4b.

Ciprofloxacin absorbance spectrum in MilliQ water at neutral pH shows a peak at 272 nm and a large absorbance band with two peaks at 316 nm and 331 nm (see Figure 4a). These absorbance features correspond to *π→π*∗ transitions of the fluorobenzene moieties and quinolone ring, respectively [54,55]. The maximum absorbance peak decrease indicates that Nexar is able to adsorb Ciprofloxacin molecules linearly with immersion time, without any change of solution pH as measured by litmus paper and as observable by the shape of absorbance spectra. After 24 h, ciprofloxacin is completely removed and measurements conducted after 48 h show no release of the adsorbed antibiotic.

As an amphoteric fluoroquinolone antibacterial agent, ciprofloxacin has a zwitterionic functional groups, i.e., the positively charged piperazine group and the negatively charged carboxyl group; these groups undergo protonation or deprotonation in acidic (pH < pKa = 5.9) or basic (pH > pKa = 8.86) solutions [56]. At pH = 6, as our experimental condition, the ciprofloxacin is in the zwitterionic form with deprotonated carboxylic groups and protonated amine, while the membrane contains sulfonic acid groups gaining a negative charge. The adsorption, thus, occurs electrostatically between negative active sites on the polymer and positive sites on antibiotic molecules. Furthermore, since both the membrane and the antibiotic contain aromatic groups the adsorption could also occur by *π*-*π* interactions.

In the NaCl 0.5 M solution (see Figure 4b), the maximum absorbance peak shifted to 278 nm and its intensity increased after the immersion of Nexar membrane. These changes are due to a decrease of solution pH from 6 to 4 when the Nexar film was immersed [57]. In particular, by reducing the pH solution, the maximum absorbance peak shifts to higher wavelength and increases in intensity [57], as we also observed in our spectra. After the shift occurred in the first minutes the absorbance peak started to decrease due to ciprofloxacin adsorption on Nexar film. Additionally, in this case, the adsorption of ciprofloxacin molecules on Nexar is linear with time, and no release was observed after 48 h. However, in this case the adsorption was not complete: according to above explained mechanism, the competition between Na ions and ciprofloxacin molecules for active sulfonic sites on polymeric membrane reduces the final efficiency.

**Figure 5:**
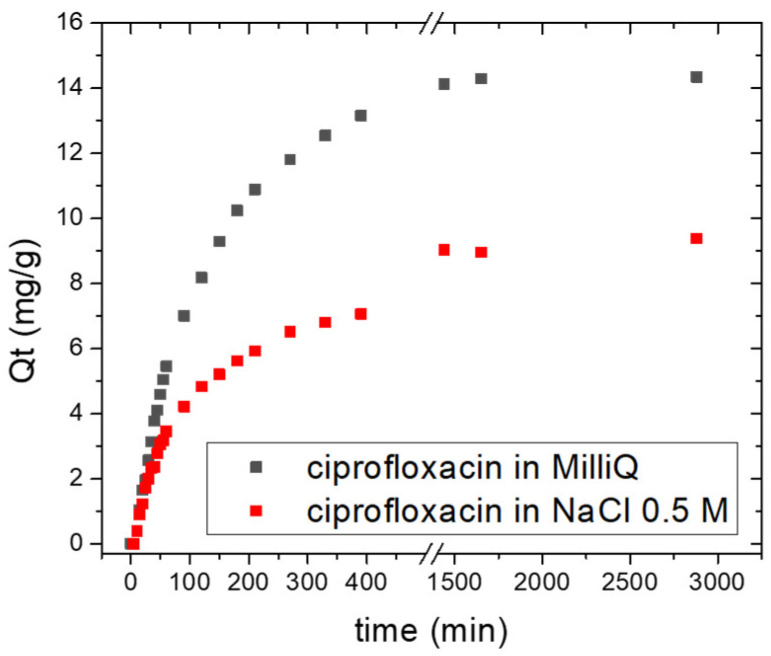
Q_t_ values calculated as a function of time for 10 ppm ciprofloxacin solution in MilliQ (black curve) or salt water (NaCl 0.5 M red curve).

The removal efficiency was estimated as the mass of antibiotic molecules adsorbed by mass unit of Nexar film for each solution, i.e., Q_t_, (mg/g). The mass of adsorbed molecules was estimated by the change of absorbance peak at 272 nm and 278 nm, respectively, for ciprofloxacin molecules in MilliQ or NaCl solutions. Figure 5 reports the Q_t_ values versus adsorption time for both solutions (these values were calculated for many adsorption times by UV-visible spectra reported in Figure S5 of Supporting Information).

For all curves, the Q_t_ values increased over time until reaching a constant final value of 14.3 and 9.4 mg/g for ciprofloxacin dispersed in MilliQ water or NaCl 0.5 M solution, respectively. Besides the final Q_t_ value, the entire Qt curve versus time for the process in NaCl solution is lower than the one in MilliQ; this could be explained taking into account the competition between Na^+^ ions and ciprofloxacin molecules for adsorption on active sites on polymeric film.

**Figure 6:**
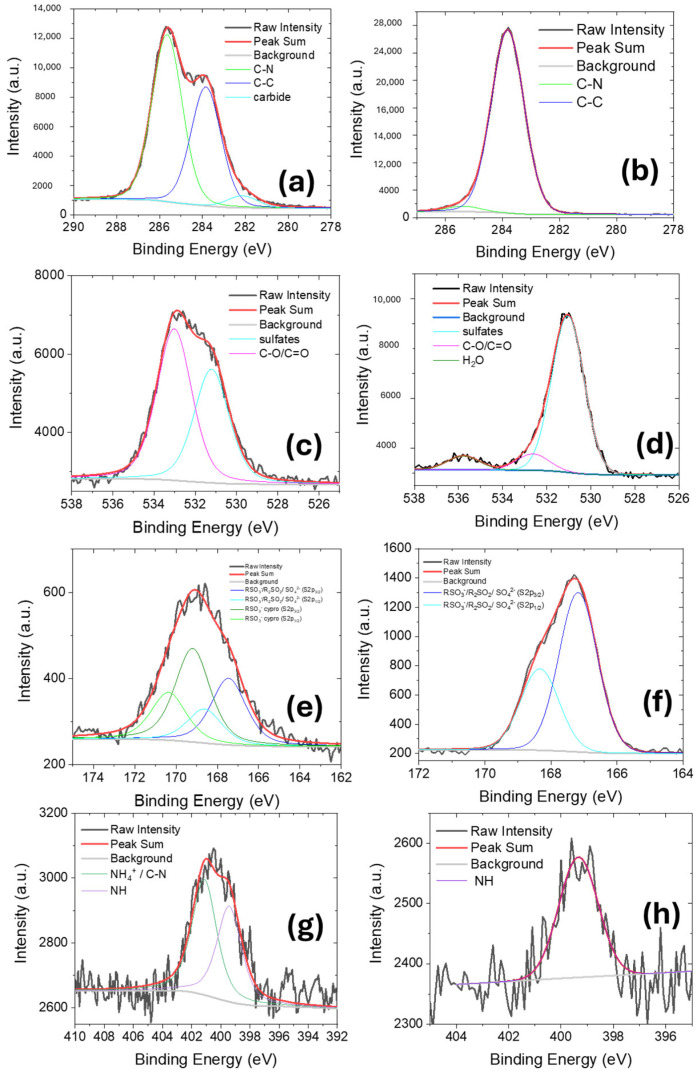
XPS spectra of C1s (**a**,**b**), O1s (**c**,**d**), S2p (**e**,**f**), and N1s (**g**,**h**) for Nexar film after adsorption in MilliQ water (on the left) and in NaCl 0.5 M (on the right) solutions. Black continuous lines indicate the acquired spectra, red lines are the fits of the spectra, grey lines are the subtracted baselines, and the other lines are the contributions obtained by peak deconvolution.

To further investigate the adsorption mechanism of ciprofloxacin, XPS spectra were acquired on Nexar films after adsorption processes in MilliQ or salty water, respectively. Survey spectra are reported in Figure S6 of Supporting Information while C1s, O1s, S2p, and N1s spectra were deconvoluted and are reported in Figure 6. By these deconvolutions, the relative amounts (%) of each species were estimated by the corresponding peak area and are reported in Table 2.

After adsorption of ciprofloxacin molecules in both MilliQ or salty water, C1s spectrum is deconvoluted into two peaks related to C-C bonds at 283.8 and C-N or C-O bonds at ~285.4 eV due to the presence of adsorbed antibiotic molecules, while the contribution present in the Nexar membrane at lower energy (carbide/vacancies) before adsorption was not detected here. In particular, the amount of C-N bonds are more relevant for the sample related to adsorption process in MilliQ: this suggests a different interaction mechanism of the antibiotic with the polymeric chains caused by the co-presence of sodium ions. Similarly, the N1s peak is deconvoluted into two contributions for the same sample related to N-H and C-N bonds at 399.4 and 401.1 eV, respectively. The last contribution is below the detection limit for sample after adsorption in salt water that reports only one contribution at 399.3 eV related to N-H bonds. Furthermore, S2p peaks deconvolution of this sample is quite similar to bare Nexar reporting only one contribution related to sulfonic acid group (RSO_3_^−^), sulfone, and sulfate. On the contrary, the S2p peak of sample after adsorption in water is deconvoluted into two contributions: the first one is related to sulfonic acid group (RSO_3_^−^), sulfone, and sulfate at 166.9 (S2p_3/2_) and 168.1 (S2p_1/2_) eV, and another contribution at higher binding energy (i.e., 168.9 and 170.1 eV) that we ascribe to the direct interaction of ciprofloxacin molecules with sulfonic acid groups of the polymeric chains. The absence of the last contribution in sample tested in salt water confirms that the interaction between ciprofloxacin and polymeric chains, in the presence of sodium ions, occurred mainly by other bonds (such as π-π) than by ionic interactions between sulfonic groups and charged antibiotic molecules. O1s spectrum for sample tested in water can be deconvoluted into two peaks having binding energy at 531.2 and 533 eV, respectively, ascribed to sulfate, i.e., O=S of SO_3_^−^ groups incorporated during the sulfonation of the polystyrene films [49], and C-O or C=O bonds present in the ciprofloxacin molecule. In the case of sample tested in salt water, another peak at 535.8 eV is reported and ascribed to gas phase water molecules [58]. In both samples, the signal of O-H reported for bare Nexar film at 530.8 eV is not detected indicating that these groups are involved in adsorption processes in both MilliQ and salty solutions.

To sum up, different adsorption mechanisms occurred in the presence of sodium ions, since these compete with antibiotic molecules for active sites on the polymeric chains and this competition forces antibiotic molecules to prefer other interactions such as π-π bonds reducing the kinetic of adsorption processes.

#### 2.2.2. Kinetic Studies

In Figure 5, three different regions according to three different slopes can be identified: (i) from 0 to 60 min where the highest slope value is observed, (ii) from 60 to 390 min the adsorption process slows down, and (iii) it reaches a plateau for the remaining time. This outlines a change of the kinetic constant of the adsorption process with time or the co-presence of different adsorption mechanisms. To deeply investigate the observed trends and adsorption mechanisms occurring, we report below a detailed kinetic analysis following three main kinetic models, i.e., the pseudo-first order (PFO), the pseudo-second order (PSO) and the intraparticle diffusion model. For this investigation, we consider the Q_t_ curves reported in Figure 5 before the plateau step, i.e., until 390 min.

During the adsorption process ciprofloxacin molecules move from the solution to the polymeric surface where they diffuse to the adsorption sites and, finally, surface adsorption and desorption reactions between adsorbate and the active groups of the adsorbent occur. Surface reactions having both chemical and physical nature are the rate determining step [59].

The models relying on this assumption are the PFO and PSO models both characterized by simplicity to manage experimental data. According to the PFO model, the controlling step of a sorption process depends on chemical reactions occurring on adsorbent surface [60]. The PFO linear expression is reported in Equation (1).

(1) $$\ln\left(Q_{e}-Q_{t}\right)=lnQ_{e}-k_{1}t$$

where k_1_ is the rate constant (min^−1^), and Q_t_ and Q_e_ are the amount of adsorbate (mg) adsorbed for mass unit of adsorbent (g) at time t or at equilibrium, respectively. The k_1_ parameter is calculated by plotting ln(Q_e_−Q_t_) versus t. Higher k1 values mean shorter times required to complete the adsorption process. Many experimental studies have confirmed that the value of the kinetic constant can be both dependent and independent of the applied operating conditions [57].

PSO equation is reported in (2):

(2) $$t/Q_{t}=\;1/(k_{2}{Q_{e}}^{2})\;+(t/Q_{e})$$

where Q_t_ and Q_e_ are the amount of adsorbate adsorbed onto the adsorbent (mg/g) at time t and at equilibrium, respectively, and k_2_ is the rate constant per min (g mg^−1^min^−1^). The Q_e_ and k_2_ parameters can be determined directly by plotting t/Q_t_ against t. In this model, the kinetic constant value strongly depends on the applied operating conditions, i.e., initial solute concentration, pH, temperature, etc. [59]. Considering only the PFO and PSO models, PSO model allows to estimate the Qt value at equilibrium leading to a more reliable description of real adsorption processes.

In order to gain a more accurate description, the diffusion phenomena, i.e., external mass transfer and internal diffusion occurring during a real adsorption process, should also be taken into account [61]. The external mass transfer could be favored by solution stirring while the internal diffusion could be described by Weber and Morris equation reported below [62].

(3) $$Q_{t}=K(t^{0.5})+C$$

where Qt (mg/g) is the amount of adsorbate adsorbed onto the adsorbent at time t, K is the rate constant (mg/g) min^0.5^, and C determines the boundary layer effect. K and C are estimated by the plotting of Qt versus t^0.5^. The value of C is linked to the boundary layer effect: the larger is the C value, the greater is the film resistance to mass transfer [42].

Figure S7 of Supporting Information reports the linear fitting of PFO, PSO and intraparticle diffusion kinetic models for ciprofloxacin adsorption in MilliQ or NaCl 0.5 M solutions, respectively, up to 390 min by 1 cm^2^ Nexar film (see Figure 4 and Figure 5). The fitting parameters are reported in Table S2 of Supporting Information. According to R^2^ values, the kinetic model that best fits experimental data for ciprofloxacin adsorption in both MilliQ water and NaCl 0.5 M is the PFO, outlining that the overall sorption process is controlled by the rate of surface reaction between ciprofloxacin molecules and active sites on Nexar surface. According to this model, the kinetic constant k_1_ and Q_e_ values were estimated by the linear fitting parameters and reported in Table 3 for both solutions. Estimated Q_e_ values are compared with the experimental Q_e_^48h^ values after 48 h of adsorption when the absorbance spectra of ciprofloxacin solutions with Nexar films did not change anymore.

**Figure 7:**
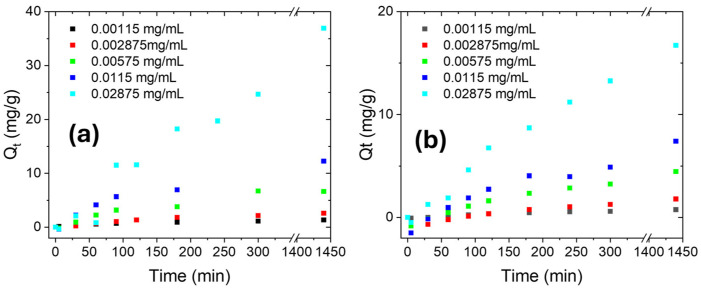
Q_t_ values calculated as a function of time at different initial ciprofloxacin concentrations in MilliQ (**a**) or NaCl 0.5 M (**b**) solutions, respectively.

K_1_ values is approximately three times higher in MilliQ than in NaCl solution, as just observed in Figure 6 and Figure 7. Similarly, Q_e_ values for ciprofloxacin adsorption in water is higher than in NaCl and both the estimated values are closer to the experimental ones. This could be explained considering that in NaCl solution, the availability of negative charged sulfonic groups on Nexar film for ciprofloxacin molecules is reduced by the competition with sodium. Indeed, this process could also be explained by diffusion model (where a R^2^ of 0.956 was found, as shown in Figure S7): when the ciprofloxacin molecules and sodium ions are both present in the solution, they compete in the adsorption process and the ability of each molecule to diffuse and reaching active sites is also a key aspect in defining the final adsorption efficiency.

This explanation is not exhaustive: even if the R^2^ values are close to 1, the fit is not satisfactory, as shown in Figure S7: two different regions of linearity can be distinguished for both the process in MilliQ and NaCl, suggesting that different mechanisms are involved.

We divide the adsorption experiments into two-time regions, i.e., the first 60 min and from 60 to 390 min. Figures S8 and S9 and Table S2 of Supporting Information report the linear fitting of experimental data and relative fitting parameters according to the three models for both ciprofloxacin adsorption in MilliQ water and NaCl 0.5 M considering two different time ranges.

For the first hour, the mechanism described by PFO and intraparticle diffusion models are predominant and quite similar in both aqueous and salty ciprofloxacin solutions: during this time, ciprofloxacin molecules are adsorbed on Nexar surface by direct interaction with surface active groups, the higher is their adsorption on the surface, the higher is the concentration gradient in solution and, consequently, their diffusion to film surface. Table 4 shows the kinetic parameters calculated according to the model with the highest R^2^ values reported in Table S2. After the first hour, the PSO model is the best fitting one for experiments conducted in saline water. For experiments conducted in MilliQ, the R^2^ values of both PFO and PSO are quite similar, but the PFO fitting reports the lowest reduced chi-square values. Indeed, when the surface active sites start to be saturated, different surface reactions can take place on the polymeric surface: chemisorption involving covalent forces and ion exchange between the adsorbent and adsorbate, and these are better described by the PSO model than by PFO [59]. Simultaneously, the depletion of adsorbate molecules in solution by surface interaction on polymeric film increases the concentration gradient at solid liquid interface, favoring the ion diffusion and this is in agreement with the high R^2^ values reported in Table S2 of Supporting Information also for the intraparticle diffusion model.

To sum up, the adsorption process is mainly determined by the surface interactions (both chemical and physical) between ciprofloxacin molecules and active sites on the polymeric film, and the nature of these interactions can be different at different adsorption times. The ion diffusion also plays a crucial role in all the investigated range as a direct consequence of ions adsorption on polymeric surface. In particular, the diffusive process has a higher weight in salty solution where sodium ions compete with ciprofloxacin for adsorption. The result of this competition is also a reduction of adsorption kinetic constant and efficiency in terms of Q_t_ for the removal of ciprofloxacin molecules in salt water.

#### 2.2.3. Isotherm Studies

In order to gain a complete understanding of adsorption phenomena, it is fundamental to get information at equilibrium conditions. Adsorption isotherms could be obtained by plotting the amount of adsorbate with respect to solute concentration and these are categorized into different types, based on the curve shape. Each of these curves describes a specific interaction mechanism between pollutants and adsorbent materials [63].

Among these, the Langmuir and Freundlich isotherms are the most used by researchers [64].

The Langmuir isotherm model regards homogeneous monolayer adsorption and relies on the assumption that adsorption and desorption rates should be equal and adsorbate-adsorbent interactions are negligible. According to the Langmuir isotherm model, all active sites should have equal affinity towards the blocked adsorbate molecules on surface [65].

Equation (4) reports the linear expression of the Langmuir model:

(4) $$1/Q_{e}=1/{(K}_{L}⨯Q_{max\;})⨯1/C_{e}+1/Q_{max\;}$$

where Ce is the adsorbate concentration in the solution at equilibrium (mg/L), Qe the solute mass adsorbed per unit adsorbent mass at equilibrium (mg/g), KL the constant of the Langmuir isotherm (L/mg), and Q_max_ is the maximum adsorption capacity (mg/g). Q_max_ and K_L_ give information on the solute affinity to the adsorbent and these are calculated from the slope and intercept of the linear fitting of Equation (4).

Multilayer adsorptions are described by the Freundlich model that defines the heterogeneity of the surface as well as the distribution of the active sites [64].

The linear Freundlich isotherm may be written as reported in Equation (5):

(5) $$logQ_{e}=\log C_{e}+logK_{F}$$

where C_e_ is the adsorbate concentration in the solution at equilibrium (mg/L), Q_e_ the solute mass adsorbed per adsorbent mass unit at equilibrium (mg/g). K_F_ and 1/n can be determined from the linear plot of logQ_e_ versus logC_e_, respectively. K_F_ is the constant of the Freundlich isotherm (L^1/n^ mg^(1−1/n)/g^), higher K_F_ values indicate higher affinity for adsorbate, and 1/n is the Freundlich exponent: the adsorption is favored when 0.1 < 1/n < 1, unfavored when 1/n > 1, or irreversible when 1/n = 1.

In order to get information on the process at equilibrium conditions, other adsorption experiments were performed by varying the initial ciprofloxacin concentration (1.15, 2.87, 5.75, 11.50, 28.75 ppm) in both MilliQ and NaCl 0.5 M solutions. The UV-visible absorbance spectra versus contact time are reported in Figure S10 of Supporting Information, showing that the absorbance peak decreases in contact with Nexar membrane versus time. The evolution of Q_t_ values versus time for each ciprofloxacin solution in both MilliQ and NaCl 0.5 M is reported in Figure 7 and the comparison of Q_t_ values after 24 h of membrane immersion in MilliQ or NaCl 0.5 M solutions is reported in Table 5.

The removal efficiency measured as Q_t_ in both MilliQ and salty solutions increased with initial ciprofloxacin concentration until reaching a constant value after 24 h. The higher the contaminant concentration is, the higher is the concentration gradient that is the driving force of its diffusion and adsorption on polymeric surface. Indeed, as described in previous section, the adsorption experiments can be divided into two time ranges, i.e., the first hour and from 60 to 390 min, underlying the coexistence of different adsorption mechanisms, i.e., direct surface interaction and diffusion. From Table 5, it is evident that all Q_t_ values are lower in NaCl solution than in MilliQ water independently of initial ciprofloxacin concentration and this is due to the competition between Na^+^ ions and ciprofloxacin molecules for adsorption on active sites of Nexar membrane.

Figure S11 of Supporting Information reports the linear fits of equilibrium data for ciprofloxacin adsorption on Nexar films in both MilliQ and salty solutions, according to the Freundlich and Langmuir models. The fitting parameters are reported in Table S3 of Supporting Information, while the adsorption parameters are reported in Table 6.

For the adsorption process conducted in aqueous solution, the model that best fits the experimental data is the Freundlich one with a R^2^ value of 0.989. Although this value is high, by using the Freundlich model for process occurring in aqueous solution, a value of 1/n equal to 1.0440 is obtained, indicating an unfavorable process. For salty solution, the best model is the Langmuir one with a R^2^ value of 0.997. in this case, a maximum adsorption capacity of −244 mg/g is estimated for the adsorption of ciprofloxacin molecules on Nexar film; this negative value has no physical meaning, indicating that the Langmuir model is not suitable for explaining the adsorption process.

Above calculations show that both Freundlich and Langmuir models are not suitable to describe our system. Indeed, the Langmuir isotherm model was initially developed to describe gas–solid interphase assuming the adsorption of a single monolayer of adsorbate [66]. On the contrary, the adsorption process described in this manuscript does not concern gas phases and it occurs at atmospheric pressure. Furthermore, we cannot assume uniform monolayer adsorption of adsorbate or solute at a specific homogenous active site; as observed by kinetic studies, in the presence of high adsorbate concentration, a rapid saturation of active sites occurred. On the other hand, the Freundlich model has been regarded as an empirical equation without physical meaning [67].

Other models usually investigated to describe contaminants adsorption from water are the SIPS [68] and Redlich–Peterson (R-P) [69] models, respectively. The SIPS isotherm combines Langmuir and Freundlich models; consequently, it fits very well gas phase processes but it has also been found to fit liquid adsorption data remarkably well, in which case the gas pressure term is replaced by the concentration of the adsorbable solute in the liquid phase [66,67]. However, the SIPS equation lacks the ability to describe the effects of important solution phase variables, such as pH and ionic strength [66,67]. For this reason it is not suitable to describe our system: indeed, the ionic strength represents the driving force of charged contaminants adsorption on charged substrate similarly to the case we report in our study. Furthermore, the SIPS equation lacks mechanistic relevance [68]: it must provide exactly the same fit of Langmuir and Freundlich models to a given set of isotherm, being the three equations mathematically equivalent.

The R–P isotherm equation incorporates additional parameters to increase the accuracy and precision of curve fitting between analytical and experimental data [70]. This isotherm balances the Langmuir and Freundlich systems, incorporating the benefits of both models but it does not allow for the description of sigmoid curve shapes [66,67,68,69,70].

All explained models are generally used for describing adsorption on microporous adsorbents (Type I isotherm) and do not consider the solvent-adsorbent interaction, that is a key aspect in processes like ours [71]. Besides the above reported models, in the case of the adsorption of solid solutes from a solution onto a solid, we can consider Giles isotherm classification (C, H, L, and S) [72] In this case, a typical S-type adsorption isotherm is observable by plotting the ratio between the amount of adsorbed contaminant at equilibrium with respect to the moles of active sites (Φ) versus solute concentrations. The non-linear shape observed in our case (i.e., sigmoidal shape) indicates the presence of a transition point resulting from the competition between the solute and the solvent for binding sites on the adsorbent.

In an ideal S-type isotherm, it is possible to identify: (i) a first region indicating that water molecules adsorption is preferred with respect to solute adsorption; (ii) a second region, associated to an equilibrium condition between water molecules and solute, where the turning point can be identified; and (iii) the last region identified by a plateau, where solute adsorption overcomes the water molecules adsorption [72].

**Figure 8:**
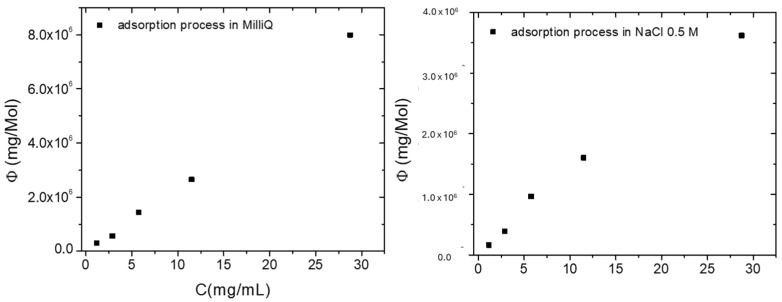
The ratio between the amount of adsorbed ciprofloxacin with respect to the moles of sulfonic groups on Nexar film (Φ), versus ciprofloxacin concentrations in MilliQ (**on the left**) or salt (**on the right**) solutions, respectively.

Figure 8 reports the Φ, i.e., the ratio between the amount of adsorbed ciprofloxacin with respect to the moles of sulfonic groups on Nexar film, versus ciprofloxacin concentrations. The amount of adsorbed ciprofloxacin molecules is measured as the difference between its concentration at equilibrium and the initial one.

The above reported plots confirm that the isotherm model valid for ciprofloxacin adsorption in MilliQ or salty solutions, respectively, are more similar to an S-type than a linear one. The first region regarding the competition between water and ciprofloxacin molecules, respectively, is slightly present only for the process in water while for the process in salty solution this step is missing; the second region within the turning point is present while for the third step, i.e., the plateau is not visible both processes. The observed and described shapes thus suggest that solute adsorption is quite dominant over water adsorption within the investigated ciprofloxacin concentration range for both processes [73].

#### 2.2.4. Regeneration of Nexar Membrane

**Figure 9:**
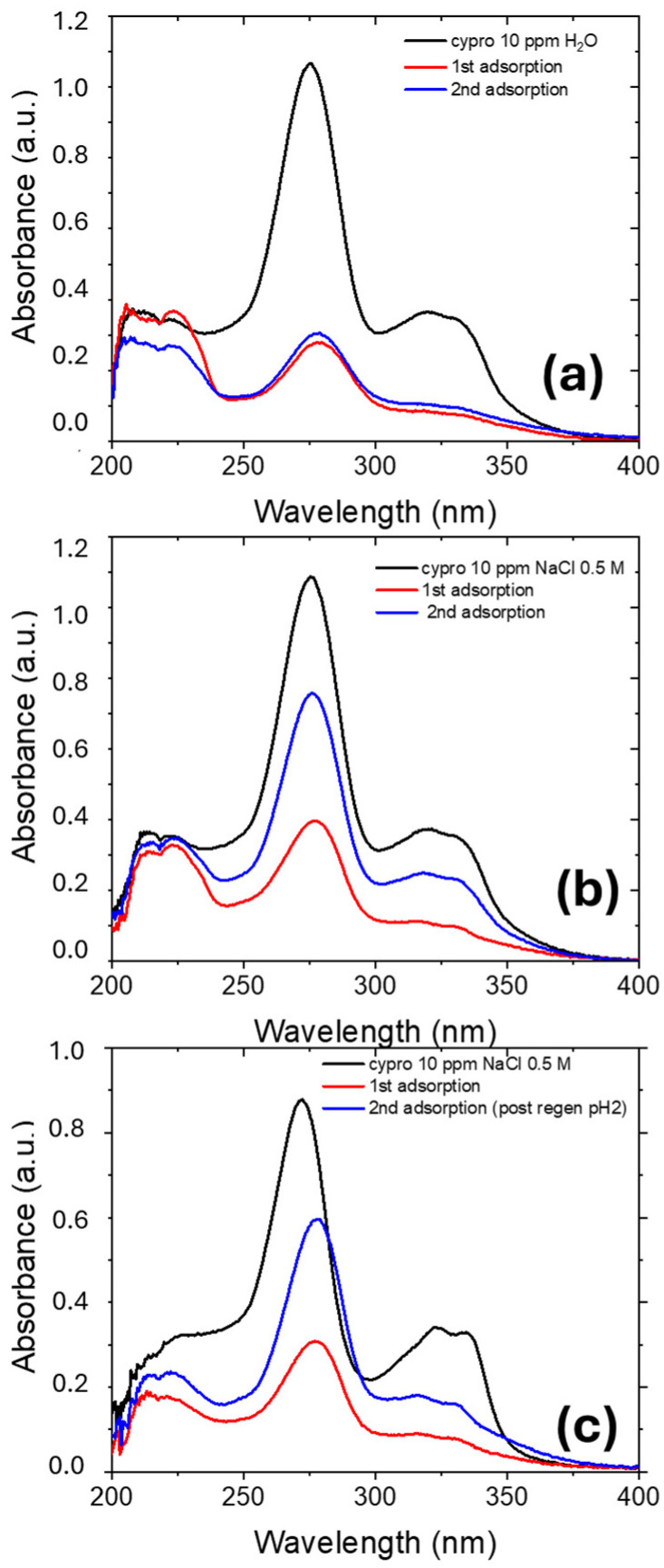
UV-visible absorbance spectra of ciprofloxacin MilliQ (**a**) and NaCl 0.5 M (**b**,**c**) solutions where Nexar films were immersed for two consecutive 24 h adsorption processes. The second adsorption processes were performed after Nexar films regeneration in MilliQ water at 50C (**a**,**b**) and in MilliQ water at pH2 and 50C (**c**).

Figure 9 reports the UV-visible absorbance spectra for ciprofloxacin aqueous solutions, where a 1 cm^2^ piece of Nexar was immersed for the first adsorption (red curve) and the second adsorption (blue curve). The last one was performed after the regeneration process described in Section 3.5.

After the regeneration process, the regenerated membrane reports the same removal ability as for the first use when the first adsorption was performed in deionized water, while if the first adsorption process is conducted in NaCl 0.5 M solution, the second adsorption is less effective. In the last case, the regeneration process was also performed in MilliQ at 50C and pH = 2, but the removal efficiency is reduced of about 50% with respect to the first adsorption. Indeed, these regeneration processes are not sufficient to remove completely adsorbed ciprofloxacin molecules or sodium ions from Nexar film. Therefore, other regeneration processes will be investigated in the case of membranes used in salt water:

This hypothesis was verified by acquiring XPS spectra on Nexar films before and after the adsorption and regeneration processes. Figure S6 of Supporting Information reports the XPS survey spectra (a) and XPS spectra of C1s (b), O1s (c), S2p (d), N1s (e), and Na1s (f) of membranes after adsorption and regeneration processes.

C, O, and S are present in the Nexar structure, while N is present only for samples that adsorbed ciprofloxacin and Na is present for samples used for adsorption processes conducted in salt water solutions. By these deconvolutions, it is possible to calculate the peak area for each element and the peak area ratio of each element with respect to the C1s peak as reported in Table 7.

As reported in Table 7, nitrogen and sodium are not present in bare Nexar film since they were introduced after the adsorption processes in water (sample A) and salty water (sample C). Sample B is the membrane A after being regenerated in MilliQ water at 50 °C: O/C and N/C ratios decreased since ciprofloxacin molecules were removed during the regeneration step.

In the case of adsorption of ciprofloxacin molecules in salty solution (sample C), both molecules and sodium ions are adsorbed at active sites of the membrane (i.e., sulfonic groups) and this competition is the reason of the lower efficiency in the removal of ciprofloxacin molecules. The differences observed in the S2p peak for antibiotics adsorption in water or salt water, suggest that adsorption could occur also by other interactions, i.e., π-π interactions with the polymeric chains, and molecules could move inside the membrane bulk. After regeneration processes in acid MilliQ water (sample D), ciprofloxacin and Na ions are not totally removed, and this explains the lower efficiency observed in the second adsorption process.

Reported results show that the Nexar adsorption capacity (calculated as mg of adsorbed ciprofloxacin on g of adsorbent) values are 14.30 and 9.40 mg/g for MilliQ or salt solution, respectively. These values are underestimated since they should be calculated by the Langmuir model that is not suitable to fit our experimental data. Anyway, these values are within the adsorption capacity values reported in the literature that range from 1.74 mg CIP/g of multi-wall carbon nanotube (MWCNT) [74] to 427.3 mg CIP/g of Hollow Co_3_S_4_ [75]. A direct comparison of adsorption properties of our material with other ones reported in the literature is not easy due to the fact that the adsorption process depends on adsorbent surface properties and many experimental parameters. The most used adsorbents are usually powders for which a direct comparison with a polymeric film is challenging since the morphology and porosity of a powder is highly different from that of a compact film, thereby affecting the transfer of contaminants to active sites. Nexar film is compact and not porous, so we do not measure a porosity and the adsorption capacity has to be measured as a function of the weight of the membrane. This is different in the case of nanomaterials or porous surfaces, where the surface/volume ratio has the main effect on the adsorption capacity. Therefore, the only way we have to compare such different materials is the adsorbate/adsorbent weight ratio (mg/g), taking into account that the adsorption conditions (temperature, pH, …) are also different for each literature work and can affect the adsorption results. We report, in Table 8, our results in comparison with some results of the literature to have an idea of the amount of material needed to adsorb a certain amount of the same contaminant (ciprofloxacin).

Concerning membranes, compact polymeric and ceramic ones have been mainly investigated for the removal of antibiotics by filtration, but these suffer from selectivity, membrane fouling, high operational costs, and are time consuming [79]. Comparing different processes as adsorption and filtration is also not possible because there is not a general rule to make experiments and a general formula to report the efficiency of the process. As an example, the contaminant amount in gram removed per area or gram of adsorbent are usually reported for adsorption processes, while in filtration process, the efficiency of the process is evaluated according to retention (%), permeability, flux, and removal efficiency (%). Among the results reported in Table 8, two examples of polymeric membranes, see refs. [41,78], can be more comparable with our kind of adsorbent; however, in both cases, the reported adsorption capacities are lower than results reported in this work by Nexar^TM^ film. Furthermore, the investigated membranes can be regenerated by washing in water or acid water, according to initial experimental conditions (i.e., MilliQ or salty water).

## 3. Materials and Methods

### 3.1. Materials

**Figure 10:**
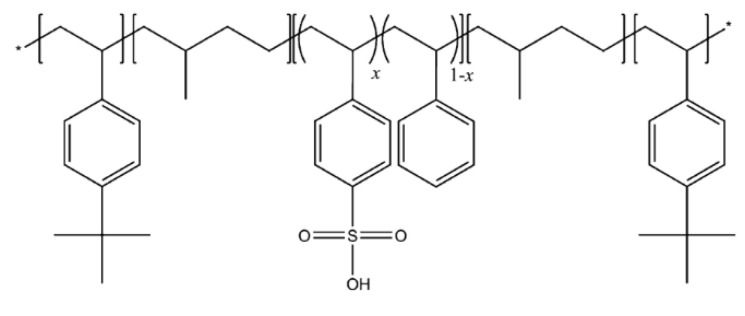
Nexar^TM^ chemical structure.

Nexar™ polymer films MD9200 (10–12 wt% polymer in a cyclohexane/heptane mixed solvent, thickness 50 μm) were provided by courtesy of Kraton Polymers LLC (Woodlands, TX, USA). The IEC value of the commercial polymer is 2.0 meq/g, corresponding to a sulfonation degree of 52 mol%. The molecular weight is 112,500 g/mol and the volume fraction (tBS-[sS:S]-HI) is 0.300-[0.226:0.208]-0.266. The copolymer is composed of blocks of tert-butyl-styrene (tBS), hydrogenated isoprene (HI) and sulfonated styrene (sS) in a molecular architecture that confers to the polymeric film mechanical resistance, flexibility, workability, and stability. The sulfonation confers to the polymer a hydrophilic character. The molecular structure of the block copolymer is reported in Figure 10.

**Figure 11:**
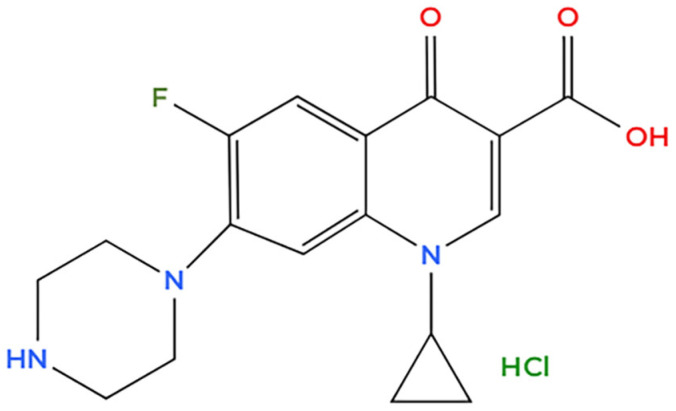
Ciprofloxacin chemical structure.

MilliQ deionized water was used for the preparation of contaminants solutions and polymeric film washing step. Ciprofloxacin hydrochloride monohydrate and sodium chloride (NaCl ≥ 99%) were purchased from Merck. Sodium chloride solution at a concentration of 0.5 M was used to investigate membrane behavior in salt water. This value is widely accepted and used in research to replicate seawater reducing the need for complex seawater handling [80,81]. Figure 11 reports the chemical structure of ciprofloxacin.

### 3.2. Water Uptake Measurement

The water uptake of the Nexar membrane was measured according to the method described by [53] and Equation (6).

Water Uptake(WU) = [(m_wet_ − m_dry_)/m_dry_] × 100 (6)

where m_wet_ corresponds to the wet mass and m_dry_ to the dry mass.

In particular, m_wet_ was measured after immersing the polymeric film in MilliQ water at room temperature for 48 h and after removing most of the free surface water by quickly wiping with a paper tissue. Afterwards, it was dried at room temperature and weighed again to measure m_dry_.

The calculated value was 141% confirming that the introduction of sulfonic groups on the polymer backbone confers it a hydrophilic character.

Before adsorption experiments, Nexar films were soaked and washed in MilliQ water at room temperature in order to remove eventual impurities, until the soaking solution stabilized at neutral pH measured by litmus paper.

### 3.3. Characterization Techniques

In order to analyze the cross section of the membrane and measure its thickness, a piece of membrane was dipped in liquid nitrogen and when it became stiff it was easily broken in two parts. These were analyzed in cross and plan by a field emission scanning electron microscope (Supra 35 FE-SEM by Zeiss, Oberkochen, Germany).

Chemical composition of the polymeric film was investigated by Fourier Transform Infrared (FT-IR) Spectroscopy, using a Jasco FT-IR-4700 spectrophotometer (Kingsgrove, NSW, Australia) equipped with an ATR (ATR-PRO ONE) with a diamond prism, and by X-ray photoelectron spectroscopy (XPS), using the PHI Genesis Multi-Technique Scanning XPS system (Physical Electronics, Inc. (PHI), Chanhassen, MN, USA), with a monochromatic Al Kα (1486.6 eV) X-ray beam and a 180° hemispherical electron energy analyzer. The Genesis system, equipped with a dual-beam charge neutralization system, allows turnkey neutralization of all types of insulating samples.

A Perkin Elmer TGA 4000 instrument (PerkinElmer Inc., Waltham, MA, USA) was used for the thermogravimetric analysis. The scans were carried out under argon flow at a flow rate of 20 mL/min. Weighed amount of Nexar (5–10 mg) were poured into an aluminum pan and subjected to heating from 100 °C to 1000 °C, at a heating rate of 10 °C min^−1^. Each sample was scanned in triplicate.

Differential scanning calorimetry (DSC) analyses were performed using the TAQ500 instrument (TA Instruments, New Castle, DE, USA) under nitrogen flow at a flow rate of 50 mL/min, from room temperature to 350 °C, with a heating rate of 5 °C/min.

### 3.4. Adsorption Experiments

The adsorption experiments were carried out at room temperature by dipping one piece of membrane of about 1 cm^2^ into 5 mL of ciprofloxacin 10 ppm in MilliQ or salt water, by adding NaCl 0.5 M. UV-VIS spectroscopic measurements were carried out using a Starline AvaSpec-2048L spectrometer equipped with an AvaLight-DH-S-BAL (by Avantes, Apeldoorn, The Netherlands) in a wavelength range between 200 and 800 nm, using wide optical window cuvettes (200–2500 nm). The adsorption efficiency was evaluated by measuring the changes in ciprofloxacin absorbance spectra with time, using the Beer–Lambert law applied to the absorbance peaks at 276 nm and 278 nm for ciprofloxacin molecules in MilliQ water and NaCl 0.5 M, respectively. The adsorption efficiency was calculated for each solution by considering the ratio between the weight (mg) of adsorbed contaminant and the membrane weight (g), defined as Q_t_ (mg/g). The adsorption experiments were conducted at neutral pH and any variation of initial pH values for different contaminants solutions was monitored during adsorption experiments using litmus paper. By these experiments, the kinetic analysis of the processes in MilliQ or salt water was carried out: the Qt values versus time were fitted with pseudo first order (PFO), pseudo second order (PO), and intraparticle diffusion (ID) kinetic models. The kinetic constants and the maximum adsorption capacities were evaluated by the plots of the linearized Equations (2)–(4). The model that has the R^2^ closest to 1 is the one that better describes the mechanism of the removal process. We also added for each fitting the value of the reduced chi-square and the lowest value indicate the best fitting. The last parameters is taken into consideration for fitting reporting quite similar values of R^2^.

Further adsorption experiments were performed by varying the initial ciprofloxacin concentration (1.15, 2.87, 5.75, 11.50, 28.75 ppm) in both MilliQ and NaCl 0.5 M solutions. UV-visible spectra were acquired after equilibrium was reached and data were fitted with Freundlich and Langmuir isotherms in MilliQ or NaCl solutions. For each experiment, the dye concentration (C_e_ ppm) and the amount of adsorbed dye for unit mass of adsorbent (Q_e_ mg/g) at equilibrium were determined. The Langmuir and Freundlich isotherm parameters were obtained from the plots of the linearized equations.

### 3.5. Membranes Regeneration

Two new Nexar pieces were tested for two consecutive 24 h adsorption processes in both MilliQ and salty water, respectively, as reported in the previous section. Before the first adsorption experiment, Nexar films were regenerated by dipping them in 5 mL MilliQ water at 50 °C in air on a hot plate for 5 h. UV-visible spectra of these solutions were acquired versus membranes dipping time to discriminate any release from them. For experiments conducted in salty water, other two regeneration procedures were investigated: membranes were dipped in 5 mL MilliQ water at pH 2 at both room temperature and 50 °C to favor the release of sodium ions. The results after both regeneration processes were identical so we report in the main text only results for regeneration process conducted at (%)C and in MilliQ at pH = 2. Spectroscopic measurements were carried out using a Starline AvaSpec-2048L spectrometer equipped with an AvaLight-DH-S-BAL (by Avantes) in a wavelength range between 200 and 800 nm, using wide optical window cuvettes (200–2500 nm). Membranes were dried at room temperature and weighed before the second adsorption process: membranes mass did not change within the balance error after the first use and regeneration process. Q_t_ values of ciprofloxacin adsorption in MilliQ and salt water for the first and second adsorption, respectively, were compared to evaluate the efficiency of the regeneration process. Ciprofloxacin stability in MilliQ or salt solution at 50 °C was also evaluated by acquiring UV-visible spectra versus time of both solutions (without Nexar films). These spectra are reported in Figure S12 of Supporting Information: no considerable changes of UV-visible spectra were observed, and the peak at 207 nm is related to NaCl.

## 4. Conclusions

The results reported in this work showed that Nexar film is a promising adsorbent for ciprofloxacin removal in both water and salt water. Chemical characterizations (both ATR FT-IR and XPS) confirm that the Nexar membrane is characterized by a copolymer pentablock chain that confers high mechanical, thermal, and chemical stability with the presence of sulfonic groups conferring to the polymer acid, hydrophilic, and adsorption properties. Furthermore, the pretreatment of the membrane with water affects its polymeric structure, improving its properties such as resistance to thermal degradation and lowering its Tg consequently to the adsorption of water molecules. The reduction of Tg indicates that the polymer chains move more freely when the membrane is immersed in water and this can positively favor contaminants adsorption.

For a ciprofloxacin concentration of 10 ppm, Nexar adsorption capacity (calculated as mg of adsorbed ciprofloxacin on g of adsorbent) values are 14.30 and 9.40 mg/g for in MilliQ or salt water, respectively. These values increase by increasing the initial contaminant concentration and are within the adsorption capacity values reported in literature.

By kinetic and isotherm analysis it is shown that the adsorption process is mainly determined by the surface interactions between ciprofloxacin molecules and active sites on the polymeric film and the contaminant adsorption is quite dominant over water adsorption within the investigated ciprofloxacin concentration range for both processes in MilliQ or salt water.

Electrostatic interactions are mainly responsible for ciprofloxacin adsorption on Nexar film in MilliQ and this explains the reduction of adsorption capacity and kinetic constant in salt solutions, since sodium ions compete with ciprofloxacin for negatively charged active sites. For both solutions, by increasing adsorption time, other interactions such as hydrophobic interactions and π-π interactions could occur also contribute to ciprofloxacin adsorption on Nexar film through its hydrophobic backbone. Furthermore, as a direct consequence of ions adsorption on polymeric surface, the diffusive process plays a crucial role in all the investigated range. In particular, it has a higher weight in salty solution where sodium ions compete with ciprofloxacin for adsorption. Adsorption mechanisms were also confirmed by XPS analysis conducted on Nexar films after adsorption processes showing different interactions mechanisms between ciprofloxacin molecules and polymeric chains in the absence of presence of sodium ions.

In conclusion, reported results confirm the possibility of using Nexar film for ciprofloxacin adsorption in water or salt water within the possibility of easily recover and regeneration of Nexar films at the end of the process. The regeneration process is easy and low cost and the membrane used in water reports a 100% efficiency in the second use and this is 50% for the membrane used in salt water. These results shed light on the possible use of Nexar film as low cost, efficient and regenerable adsorptive membrane for water purification purposes.

## Figures and Tables

**Table 1:** The relative amounts (%) of each species present in Nexar membrane, calculated by peak deconvolutions reported in Figure 1.

Peak	Species	Binding Energy (eV)	Relative Amount (%)
C1s	Carbide	282.3	14.3
	C-C	283.9	85.7
S2p	RSO_3_^−^/R_2_SO_2_/SO_4_^2−^ (S2p_3/2_)	167.5	66.2
	RSO_3_^−^/R_2_SO_2_/SO_4_^2−^ (S2p_1/2_)	168.6	33.8
O1s	hydroxides	530.8	56.6
	sulfates	531.8	43.4

**Table 2:** The relative amounts (%) of each species present in Nexar membrane, calculated by peak deconvolutions reported in Figure 6.

Peak	Species	Nexar		Nexar Post Adsorption in MilliQ		Nexar Post Adsorption in Salt Water	
		Binding Energy (eV)	Relative Amount (%)	Binding Energy (eV)	Relative Amount (%)	Binding Energy (eV)	Relative Amount (%)
C1s	Carbide	282.3	14.3	282.1	4.0	--	--
	C-C	283.9	85.7	283.8	39.9	283.8	97.0
	C-N/C-O	--	--	285.6	56.1	285.4	3.0
O1s	hydroxides	530.8	56.6	--	--	--	--
	sulfates	531.8	43.4	531.2	42.8	531.0	84
	C-O/C=O	--	--	533.0	57.2	532.6	9
	Gas phase H_2_O	--	--	--	--	535.8	7
S2p	RSO_3_^−^/R_2_SO_2_/SO_4_^2−^ (S2p_3/2_)	167.5	66.2	166.9	21.2	167.2	66.2
	RSO_3_^−^/R_2_SO_2_/SO_4_^2−^ (S2p_1/2_)	168.6	33.8	168.1	10.2	168.3	33.8
	RSO_3_^−^ cypro (S2p_3/2_)	--	--	168.9	45.0	--	--
	RSO_3_^−^ cypro (S2p_1/2_)	--	--	170.1	23.0	--	--
N1s	NH (organic)	--	--	399.4	45.3	399.3	100
	NH_4_^+^/C-N (organic)	--	--	401.1	54.7	--	--

**Table 3:** Pseudo first order (PFO) parameters for ciprofloxacin adsorption on Nexar film in both MilliQ and NaCl 0.5 M solutions.

Ciprofloxacin Solution	R^2^	k_1_ (min^−1^)	Q_e_ (mg/g)	Q_e_^48h^(mg/g)
MilliQ	0.995	−0.0064 ± 0.0001	13.95	14.30
NaCl 0.5 M	0.959	−0.0024 ± 1.1314	8.62	9.40

**Table 4:** Kinetic parameters of the PFO and PSO models for ciprofloxacin adsorption in MilliQ and NaCl 0.5 M solutions, respectively, for the two time ranges. The indicated models provide the best fitting for each contaminant and each time range, as extracted from Table S2.

Process Time	Model	Ciprofloxacin in MilliQ			Ciprofloxacin in NaCl 0.5 M		
		R^2^	k_1_ (min^−1^)	Q_e_ (mg/g)	R^2^	k_1_ (min^−1^)	Q_e_ (mg/g)
0–60 min	PFO	0.998	−0.0092	15.39	0.994	−0.0047 ± 0.0001	9.34
		R^2^	k_2_ (min^−1^)	Q_e_ (mg/g)	R^2^	k_2_ (min^−1^)	Q_e_ (mg/g)
60–390 min	PSO	0.999	0.0004	17.80	0.999	0.0011	8.94

**Table 5:** Comparison of Q_t_ values after 24 h of membrane immersion in MilliQ or NaCl 0.5 M solutions.

Ciprofloxacin Initial Concentration (ppm)	Q_e_ (mg/g) in MilliQ	Q_e_ (mg/g) in NaCl 0.5 M
1.15	1.34	0.75
2.87	2.60	1.78
5.75	6.62	4.46
11.50	12.25	7.40
28.75	36.93	16.73

**Table 6:** Fitting parameters of Langmuir and Freundlich isotherm for ciprofloxacin adsorption on Nexar films in both MilliQ and NaCl 0.5 M solutions.

Ciprofloxacin Solutions	Langmuir			Freundlich		
	Q_max_ (mg/g)	K_L_ (L/mg)	R^2^	1/n	K_F_ (L^1/n^ mg^(1−1/n)^/g)	R^2^
MilliQ	61	−59.84	0.978	1.0440	1.0247	0.989
NaCl 0.5 M	−244	244.65	0.997	0.9737	0.6825	0.991

**Table 7:** Peak area ratios between each element and the C1s peak for Nexar membranes after adsorption and regeneration processes. These values were calculated by using peaks area reported in Figure S6 of Supporting Information. “A” is the membrane after adsorption of ciprofloxacin 10 ppm in MilliQ, “B” stands for membrane “A” after regeneration in MilliQ at 50 °C, “C” stands for membrane after adsorption of ciprofloxacin 10 ppm in NaCl 0.5 M; “D” is the membrane after adsorption of ciprofloxacin 10 ppm in NaCl 0.5 M and regenerated at 50 °C in acid MilliQ (pH = 2), respectively. XPS spectra of bare Nexar film is added as comparison.

Sample	Element Peak Area Ratio			
	O/C	S/C	N/C	Na/C
Nexar	0.17	0.03	--	--
A	0.99	0.08	0.10	-
B	0.41	0.06	0.06	0.03
C	0.31	0.06	0.01	0.09
D	0.42	0.04	0.01	0.04

**Table 8:** Adsorption capacity of some adsorbents for ciprofloxacin removal.

Adsorbents	Adsorption Capacity (mg/g)	References
Nexar (MilliQ)	14.30	This work
Nexar (NaCl 0.5 M)	9.40	This work
MWCNTs	1.74	[74]
Fe_3_O_4_ NPs	24	[76]
Hollow Co_3_S_4_ NPs	427.3	[75]
Powdered activated carbon	13.6	[77]
Montmorillonite	0.60	[74]
Modified Montmorillonite	1.67	[74]
Alumina	1.15	[74]
PES/ZrP	8.9	[41]
Polyethylene microplastic	2–3	[78]

## Data Availability

The original contributions presented in this study are included in the article/Supplementary Materials. Further inquiries can be directed to the corresponding authors.

## Supplementary Materials

The following supporting information can be downloaded at: https://www.mdpi.com/article/10.3390/molecules30153275/s1, Figure S1. SEM image of Nexar film cross section; Figure S2: FT-IR spectra of Nexar film in the range 4000–500 cm^−1^; Figure S3. XPS survey spectra of Nexar film; Figure S4. UV-visible absorbance spectra (on the left) and relative calibration curves (on the right) of ciprofloxacin solutions within a concentration range between 1.15 and 28.75 ppm. For the calibration curve (on the right) the absorbance value at 278 nm was reported versus the initial antibiotic concentration; Figure S5. UV-visible absorbance spectra versus time obtained for ciprofloxacin solutions prepared using MilliQ water (a) and simulated sea water (b) where Nexar film was immersed; Figure S6. XPS survey spectra (a) and XPS C1s (b), O1s (c), S2p (d), N1s (e), and Na1s (f) spectra acquired on membranes after adsorption and regeneration processes. Nexar indicates the initial membrane, while A stands for membrane after adsorption of cipro 10 ppm in MilliQ, B stands for membrane A after regeneration in MilliQ at 50 °C, C stands for membrane after adsorption of cipro 10 ppm in NaCl 0.5 M, D stands for membrane after adsorption of cipro 10 ppm in NaCl 0.5 M and regenerated at 50 °C in acid MilliQ (pH = 2), respectively. The intensity of sample B was multiplied by five times to be more visible. Figure S7: Linear fitting of PFO, PSO and diffusion kinetic models for ciprofloxacin adsorption in MilliQ or NaCl 0.5 M solutions, respectively, up to 390 min; Figure S8. Linear fitting of PFO, PSO, and diffusion kinetic models for ciprofloxacin adsorption in MilliQ or NaCl 0.5 M solutions, respectively, in the time range 0–60 min; Figure S9. Linear fitting of PFO, PSO and diffusion kinetic models for ciprofloxacin adsorption in MilliQ or NaCl 0.5 M solutions, respectively, in the time range 60–390 min; Figure S10. UV-visible absorbance spectra versus time of ciprofloxacin in MilliQ (on the left) or NaCl 0.5 M (on the right) solutions, respectively, where the Nexar membrane was immersed. Ciprofloxacin initial concentration values are between 1.15 ppm and 28.75 ppm; Figure S11. Freundlich (a,c) and Langmuir (b,d) plots for ciprofloxacin solutions in MilliQ (a,b) water and NaCl 0.5 M (c,d) solutions, respectively; Figure S12. UV-visible absorbance spectra of ciprofloxacin MilliQ (a) and NaCl 0.5 M (b) solutions at 50 °C versus time. Table S1. Fitting parameters of PFO, PSO and diffusion kinetic models for ciprofloxacin (10 ppm) adsorption in MilliQ or NaCl 0.5 M solutions, respectively, up to 390 min; Table S2. Fitting parameters of PFO, PSO, and diffusion kinetic models for ciprofloxacin adsorption in MilliQ or NaCl 0.5 M solutions, respectively, in two different time regions, i.e., 0–60 min and 60–390 min; Table S3. Fitting parameters of Langmuir and Freundlich isotherms for ciprofloxacin adsorption on Nexar films in both MilliQ and NaCl 0.5 M solutions.
