# Supplementary material for: Effective Ciprofloxacin Removal from Deionized and Salt Water by Sulfonated Pentablock Copolymer (NexarTM)
Source: Molecules. 2025 Aug 5;30(15):3275. doi: 10.3390/molecules30153275 (PMC12348623; doi:10.3390/molecules30153275)
Supplement: Supplementary file 1 [file molecules-30-03275-s001.zip › molecules-3679922-supplementary.pdf]

Effective Ciprofloxacin Removal from Deionized and Salt Water by Sulfonated Pentablock Copolymer (Nexar™)

Simona Filice [1,*], Simona Crispi [1], Viviana Scuderi [1], Daniela Iannazzo [2], Consuelo Celesti [2] and Silvia Scalese [1,*]

[1] Consiglio Nazionale delle Ricerche, Istituto per la Microelettronica e Microsistemi (CNR-IMM), Ottava Strada n.5, I-95121 Catania, Italy; simona.crispi@cnr.it (S.C.); viviana.scuderi@imm.cnr.it (V.S.)

[2] Department of Engineering, University of Messina, Contrada Di Dio, I-98166 Messina, Italy; daniela.iannazzo@unime.it (D.I.); consuelo.celesti@unime.it (C.C.)

* Correspondence: simona.filice@cnr.it (S.F.); silvia.scalese@imm.cnr.it (S.S.)

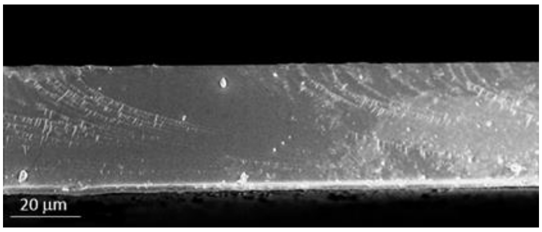

Figure S1. SEM image of Nexar film cross section.

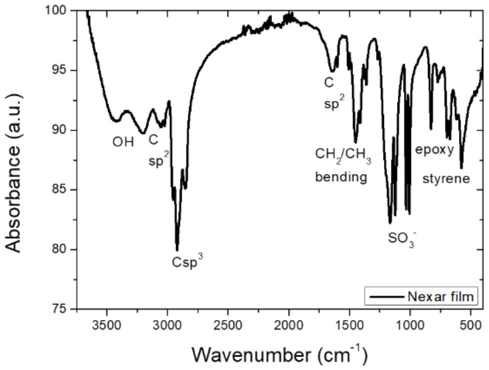

Figure S2: FT-IR spectra of Nexar film in the range 4000-500 cm^{-1}.

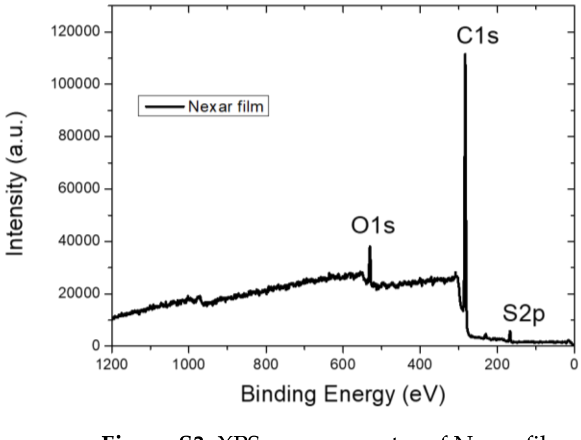

Figure S3. XPS survey spectra of Nexar film.

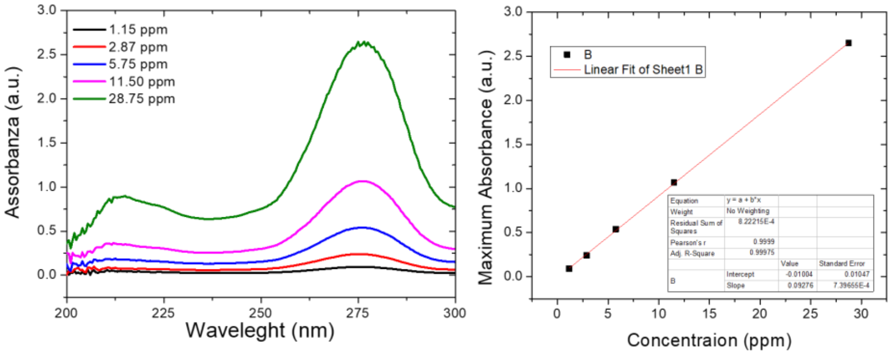

Figure S4. UV-Visible absorbance spectra (on the left) and relative calibration curves (on the right) of ciprofloxacin solutions within a concentration range between 1.15 to 28.75 ppm. For the calibration curve (on the right) the absorbance value at 278 nm was reported versus the initial antibiotic concentration.

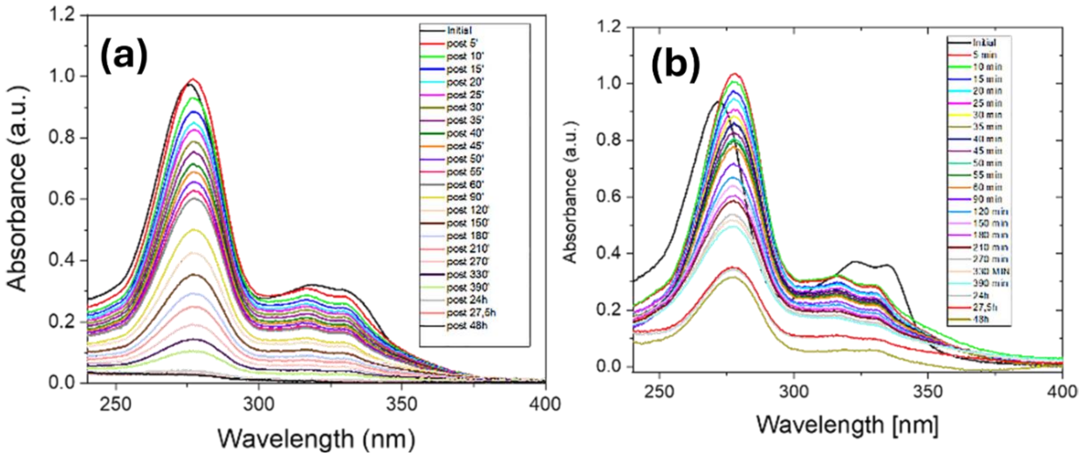

Figure S5. UV-Visible absorbance spectra versus time obtained for Ciprofloxacin solutions prepared using MilliQ water (a) and simulated sea water (b) where Nexar film was immersed.

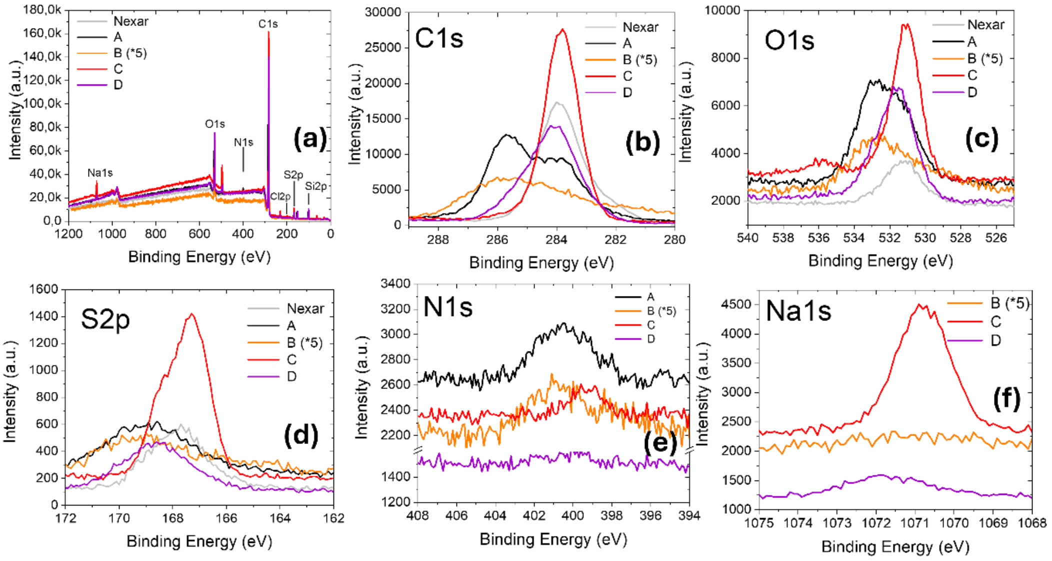

Figure S6. XPS survey spectra (a) and XPS C1s (b), O1s (c), S2p (d), N1s (e) and Na1s (f) spectra acquired on membranes after adsorption and regeneration processes. Nexar indicates the initial membrane, while A stands for membrane after adsorption of cipro 10 ppm in MilliQ, B stands for membrane A after regeneration in MilliQ at 50°C, C stands for membrane after adsorption of cipro 10 ppm in NaCl 0.5 M, D stands for membrane after adsorption of cipro 10 ppm in NaCl 0.5 M and regenerated at 50°C in acid MilliQ (pH=2), respectively. The intensity of sample B was multipled by 5 times to be more visible.

Table S1. Fitting parameters of PFO, PSO and diffusion kinetic models for ciprofloxacin (10 ppm) adsorption in MilliQ or NaCl 0.5 M solutions, respectively, up to 390 minutes.

Cyprofloxacin solution	Kinetic model	R^2	Reduced chi-sqr	SLOPE	INTERCEPT
MilliQ	PFO	0.995	0.0031	-0.0064 ± 0,0001	2.6358 ± 0,0156
	PSO	0.468	0.3043	0.0538 ± 0.0125	8.4845 ± 1.8701
	DIFF	0.970	0.0447	0.8003 ± 0.0312	-1.33641 ± 0.3140
NaCl 0.5 M	PFO	0.959	0.0033	-0.0024 ± 1.1314	2.1545 ± 0.0167
	PSO	0.942	8.7935	0.1048 ± 0.0061	13.2320 ± 0.2088
	DIFF	0.956	0.2062	0.4160 ± 0.0202	-0.2692 ± 0.0495

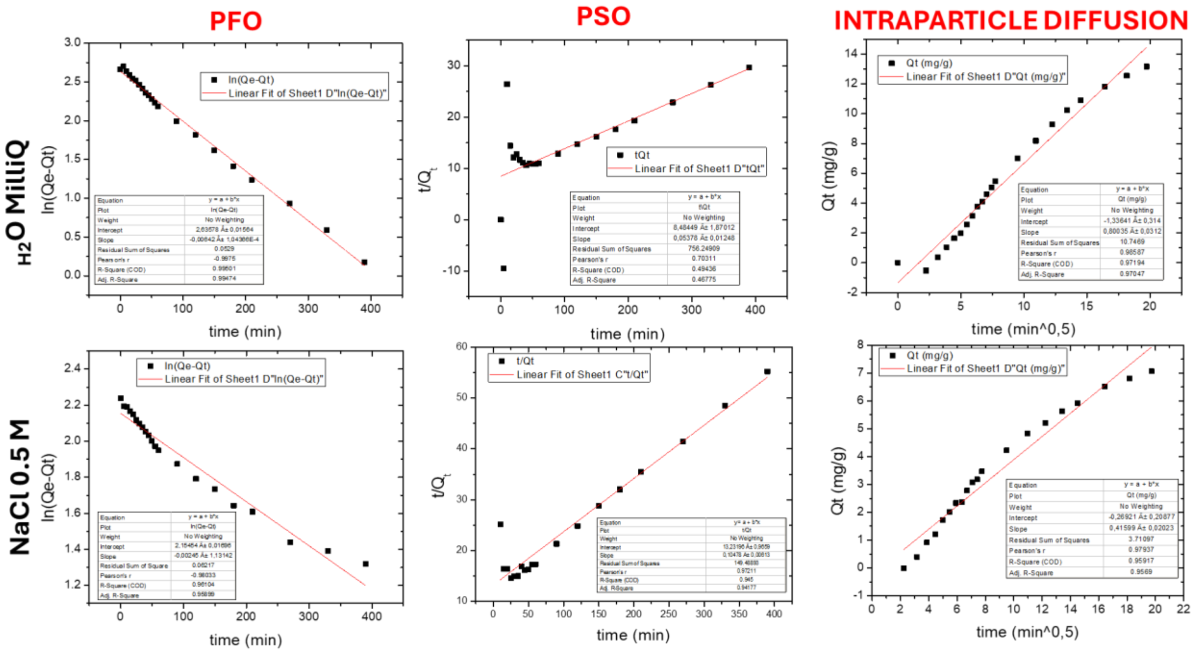

Figure S7: Linear fitting of PFO, PSO and diffusion kinetic models for ciprofloxacin adsorption in MilliQ or NaCl 0.5 M solutions, respectively, up to 390 minutes.

Table S2. Fitting parameters of PFO, PSO and diffusion kinetic models for ciprofloxacin adsorption in MilliQ or NaCl 0.5 M solutions, respectively, in two different time regions i.e. 0-60 minutes and 60-390 minutes.

Ciprofloxacin solutions	Model	Process Times							
		0-60 min				60-390 min			
		R^2	Reduced chi-sqr	SLOPE	INTERCEPT	R^2	Reduced chi-sqr	SLOPE	INTERCEPT
MilliQ	PFO	0.998	0.00006	-0.0092 ± 0.0001	2.7339 ± 0.0048	0.998	0.0007	-0.0060 ± 0.0001	2.5265 ± 0.0191
	PSO	-0.093	67.250	0.0340 ± 0.1372	10.0064 ± 5.0472	0.999	0.0240	0.0562 ± 0.0005	7.6936 ± 0.1115
	DIFF	0.992	0.028	1.0901 ± 0.0291	-3.1754 ± 0.1658	0.968	0.2128	0.6398 ± 0.0408	1.1160 ± 0.5777
NaCl 0.5 M	PFO	0.994	0.00005	-0.0047 ± 0.0001	2.2349 ± 0.0037	0.967	0.0013	-0.0019 ± 1.3088	2.0085 ± 0.2859
	PSO	0.007	8.1710	-0.0563 ± 0.0545	18.9207 ± 2.0935	0.999	0.1105	0.1118 ± 0.0012	11.6238 ± 2.8935
	DIFF	0.994	0.0078	0.6436 ± 0.0147	-0.3830 ± 0.0461	0.977	0.0228	0.2771± 0.0161	1.7910 ± 0.2367

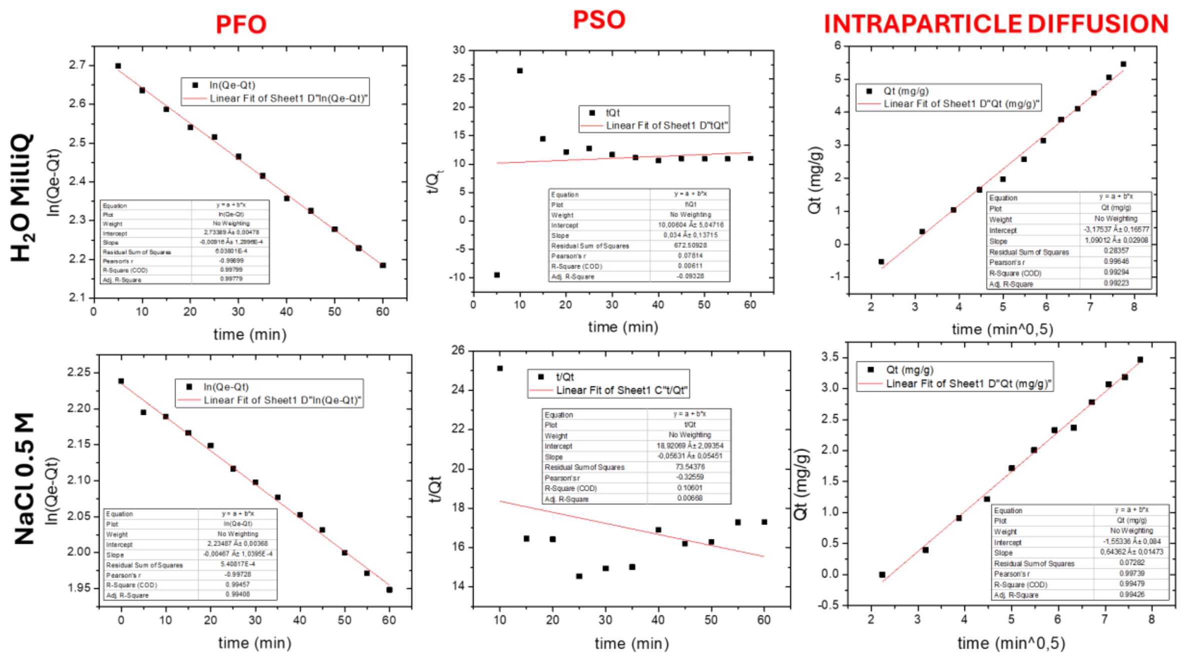

Figure S8. Linear fitting of PFO, PSO and diffusion kinetic models for ciprofloxacin adsorption in MilliQ or NaCl 0.5 M solutions, respectively, in the time range 0-60 minutes.

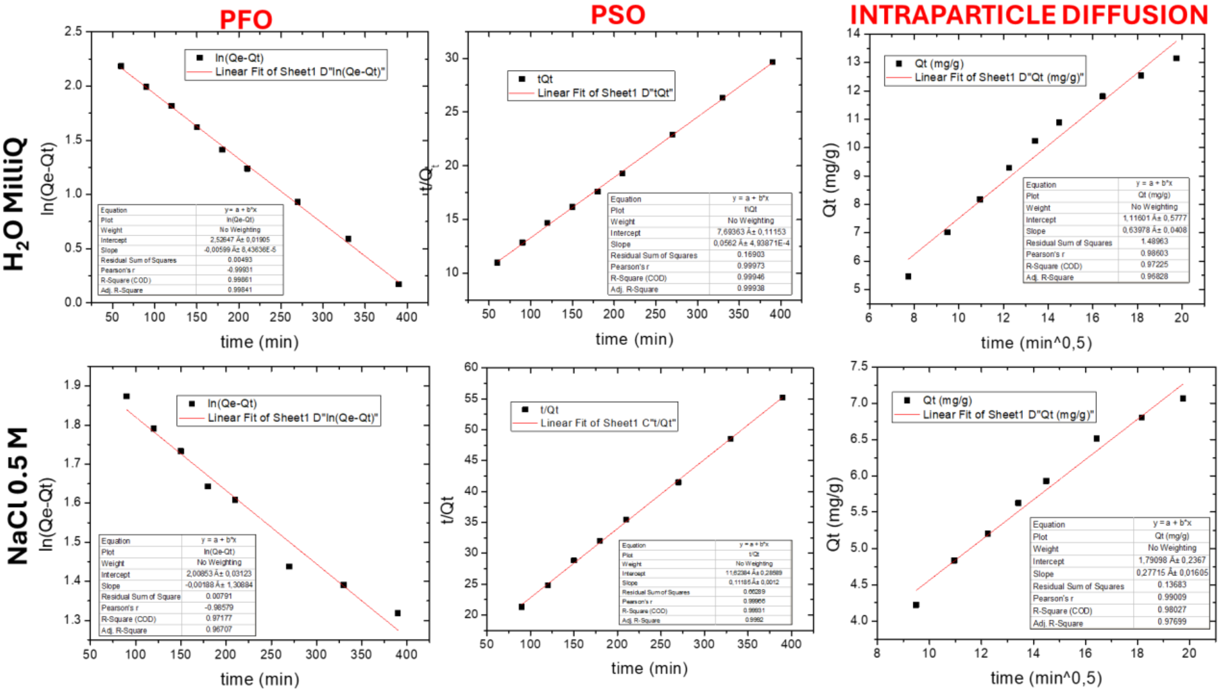

Figure S9. Linear fitting of PFO, PSO and diffusion kinetic models for ciprofloxacin adsorption in MilliQ or NaCl 0.5 M solutions, respectively, in the time range 60-390 minutes.

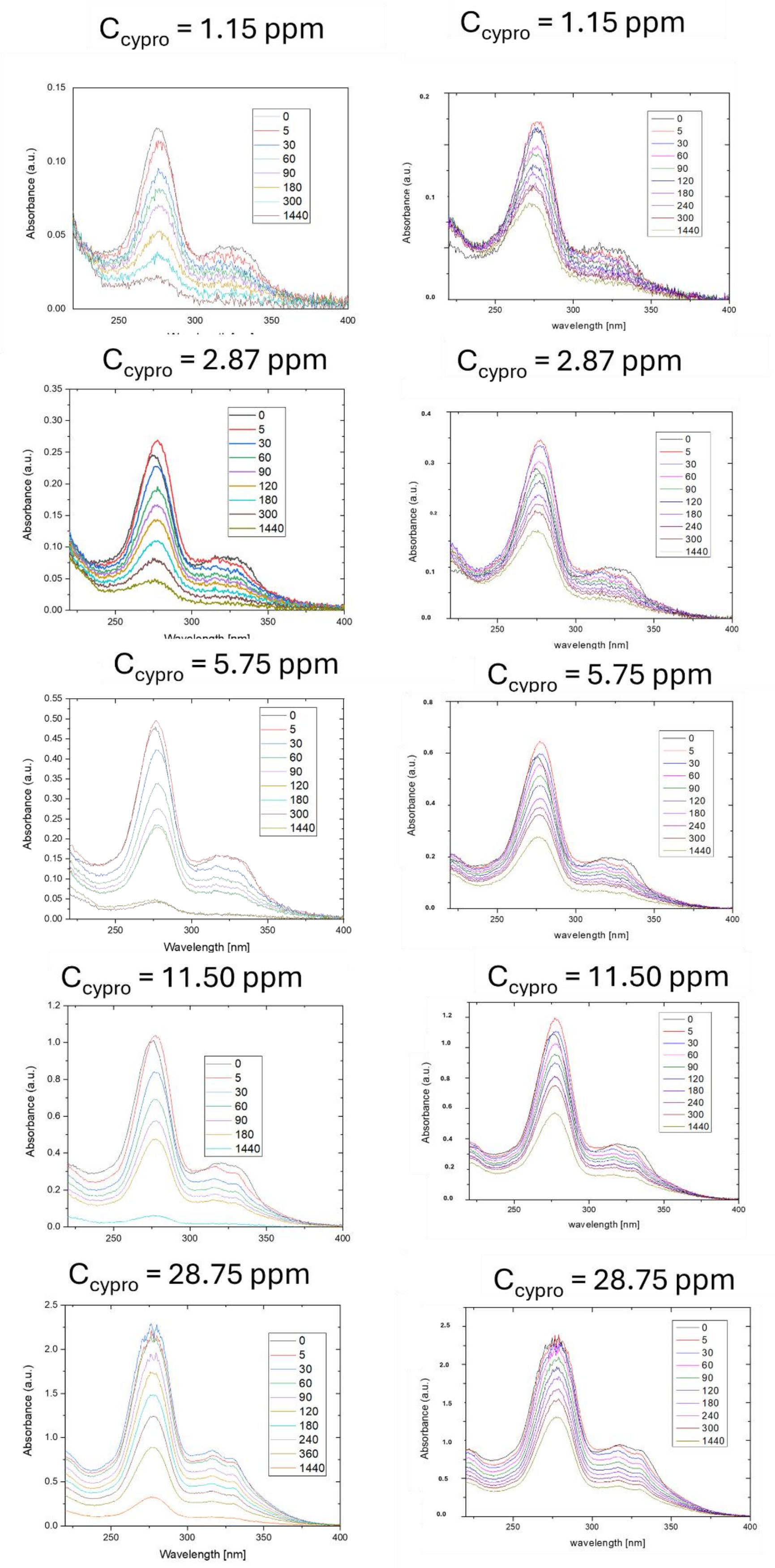

Figure S10. UV-Visible absorbance spectra versus time of ciprofloxacin in MilliQ (on the left) or NaCl 0.5 M (on the right) solutions, respectively, where Nexar membrane was immersed. Ciprofloxacin initial concentration values are between 1.15 ppm and 28.75 ppm.

Table S3. Fitting parameters of Langmuir and Freundlich isotherms for ciprofloxacin adsorption on Nexar films in both MilliQ and NaCl 0.5 M solutions.

**Cyprofloxacin solutions**	**Langmuir**				**Freundlich**			
	**INTERCEPT**	**SLOPE**	**R^2**	**Reduced chi-sqr**	**INTERCEPT**	**SLOPE**	**R^2**	**Reduced chi-sqr**
**MilliQ**	0.0165 ± 0.0277	0.8645 ± 0.0647	**0.978**	0.00192	0.0244 ± 0.1149	1.0440 ± 0.0555	**0.989**	0.01892
**NaCl 0.5 M**	-0.0041±0,0183	1.5410±0,0426	**0.997**	0.00083	-0.3820±0,0978	0.9737±0,0472	**0.991**	**0.01371**

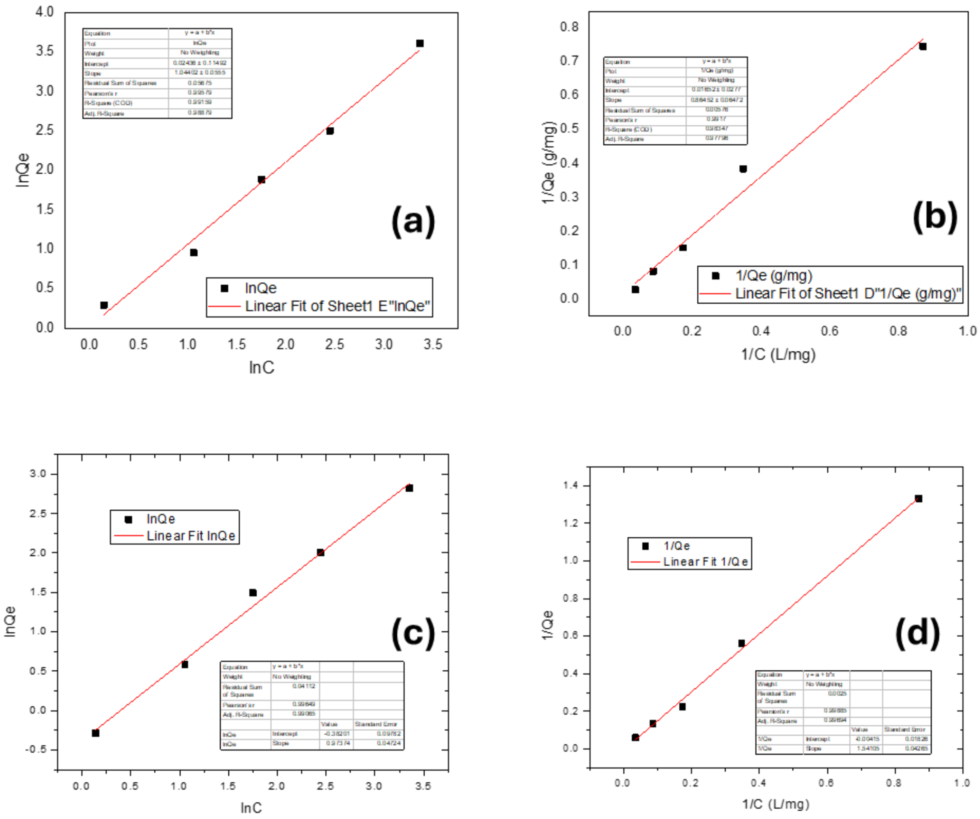

Figure S11. Freundlich (a,c) and Langmuir (b,d) plots for ciprofloxacin solutions in MilliQ (a,b) water and NaCl 0.5 M (c,d) solutions, respectively.

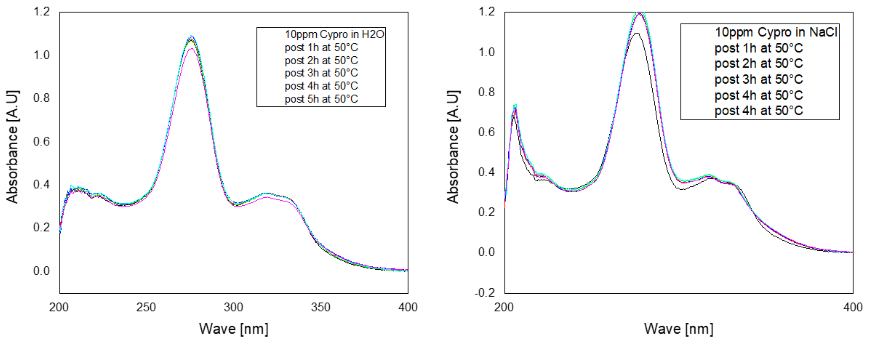

Figure S12. UV-Visible absorbance spectra of Ciprofloxacin MilliQ (a) and NaCl 0.5 M (b) solutions at 50C versus time.
